# Enzymatic and Bioinspired Systems for Hydrogen Production

**DOI:** 10.3390/ijms24108605

**Published:** 2023-05-11

**Authors:** Linda Leone, Gianmattia Sgueglia, Salvatore La Gatta, Marco Chino, Flavia Nastri, Angela Lombardi

**Affiliations:** Department of Chemical Sciences, University of Naples Federico II, 80126 Naples, Italy; gianmattia.sgueglia@unina.it (G.S.); salvatore.lagatta@unina.it (S.L.G.); marco.chino@unina.it (M.C.); flavia.nastri@unina.it (F.N.)

**Keywords:** hydrogen, hydrogenases, hydrogen-evolving catalysts, bioinspired catalysis, artificial enzymes, small-molecule complexes, sustainable energy production, biofuel cells, photocatalytic hydrogen

## Abstract

The extraordinary potential of hydrogen as a clean and sustainable fuel has sparked the interest of the scientific community to find environmentally friendly methods for its production. Biological catalysts are the most attractive solution, as they usually operate under mild conditions and do not produce carbon-containing byproducts. Hydrogenases promote reversible proton reduction to hydrogen in a variety of anoxic bacteria and algae, displaying unparallel catalytic performances. Attempts to use these sophisticated enzymes in scalable hydrogen production have been hampered by limitations associated with their production and stability. Inspired by nature, significant efforts have been made in the development of artificial systems able to promote the hydrogen evolution reaction, via either electrochemical or light-driven catalysis. Starting from small-molecule coordination compounds, peptide- and protein-based architectures have been constructed around the catalytic center with the aim of reproducing hydrogenase function into robust, efficient, and cost-effective catalysts. In this review, we first provide an overview of the structural and functional properties of hydrogenases, along with their integration in devices for hydrogen and energy production. Then, we describe the most recent advances in the development of homogeneous hydrogen evolution catalysts envisioned to mimic hydrogenases.

## 1. Hydrogen: A Fuel for the Future

Over the last century, modern society has reached a peak of energy consumption, required to withstand the technological progress and population growth. Fossil fuel is currently the primary source of energy, despite its impact on the environment and limited supply. Hence, developing sustainable alternatives is definitely the most urgent challenge for humanity [1]. Hydrogen (H_2_) represents a promising fuel to manage the existing energetic crisis, owing to its exceedingly high energy density (~120 MJ/kg), which surpasses carbon-based sources as methane and natural gas (~50 MJ/kg) [2]. Most importantly, hydrogen can be converted into electricity in a fuel cell or combusted into an engine, producing only water as the byproduct. Despite no carbon products being formed through its exploitation, considering hydrogen as a clean fuel is not straightforward, since several issues need to be addressed concerning the environmental impact of its production, transportation, and storage [3]. Being gaseous in ambient conditions, hydrogen needs to be stored under high pressure or liquified. These processes require additional energy, which may not come from renewable sources. Furthermore, despite being the most abundant element on Earth, hydrogen is readily available only in combination with other elements, but not in the elementary form. Currently, about ~96% of H_2_ is produced on an industrial scale from nonrenewable fossil fuels, mostly via the steam-reforming process, where water steam reacts with hydrocarbons to give H_2_ and CO_2_ [4]. The significant emissions of greenhouse gases, coupled to excessive energy requirement, are some of the inherent limitations of these methods. Therefore, there is an urgent need to design H_2_ production systems that are both effective and sustainable.

Electrolysis of water can be considered as a sustainable alternative, especially when using solar or wind energy as source of electric power [5,6]. However, this method is still not economically viable, due to the high energy consumption and slow hydrogen evolution rates. To increase the efficiency of such a process, much research has been devoted to developing proficient and low-cost electrocatalysts for hydrogen evolution. Biological catalysts are the most environmentally friendly alternatives, as they usually operate under mild reaction conditions and typically do not produce carbon-containing byproducts. Biological pathways include those exploiting wastewater or organic solid waste as substrates [7,8]. Among them, anaerobic fermentation is the most studied, involving the conversion of organic molecules from waste by microorganisms such as anoxic bacteria [9,10,11]. Photobiological processes are, in principle, even more advantageous, as they involve the use of photosynthesis and the natural water-splitting process, in which the microbes absorb energy from the sun and evolve H_2_ directly from water, without an intermediate biomass stage [12,13]. Such a process requires the synergic work of a photosynthetic system and a hydrogenase enzyme, which are both found in cyanobacteria and green algae [14,15]. Albeit promising from a sustainability perspective, photobiological H_2_ production is limited by the low overall efficiency of natural photosynthesis. The challenge of converting solar energy more efficiently than natural organisms has inspired the development of hybrid systems comprising the use of synthetic photosensitizers coupled to hydrogenase enzymes [16,17,18,19]. However, scaling up these systems has proven to be very difficult due to the need for large-scale expression and purification of the enzymes. Furthermore, a significant drawback is the high O_2_ sensitivity of hydrogenases, particularly algal [FeFe]-hydrogenases [20].

A possible strategy to overcome the limitations associated with the low availability of hydrogenases is to build entirely artificial systems, which require the construction of robust, efficient, and easily viable mimetics of hydrogenase enzymes [21]. Following this path, a wide variety of molecular and biomolecular catalysts have been developed over the last decades, displaying either electrochemical or light-driven hydrogen-evolving activity [22]. In this review, we first describe the structural and functional properties of hydrogenases and summarize the most recent advances in the development of devices for hydrogen and energy production based on these natural enzymes. Then, we provide an overview of the artificial homogeneous catalysts envisioned to mimic hydrogenase function, including small-molecule, peptide-based, and protein-based analogues.

## 2. Natural Hydrogenases

Hydrogenases are a class of widely distributed metalloenzymes able to catalyze both hydrogen production from protons and electrons and the reverse reaction, i.e., oxidation of molecular hydrogen to protons (H_2_ ⇌ 2H^+^ + 2e^−^) [23]. The relevance of this process to hydrogen synthesis has made them privileged biomolecular systems to study, with the initial goal of obtaining catalysts and devices that could be used for hydrogen evolution and, ultimately, for sustainable energy production. In biology, hydrogenases are required by some organisms, such as archaea, bacteria, and green algae, for H_2_-based respiration and are involved in maintaining the redox balance inside cells, by using reducing equivalents to reduce protons where necessary. Hydrogenases are usually located in the cytoplasm or periplasm, in either membrane-bound or soluble form. Several hydrogenases, known as regulatory hydrogenases, almost devoid of catalytic activity, are instead devoted to simply sensing the presence of hydrogen and precisely controlling the expression of genes coding for active hydrogenases and other associated proteins [24]. Most of these metalloenzymes are sensitive to the presence of molecular oxygen [25], which inactivate them via different mechanisms, depending on the specific hydrogenase type. This weakness is probably inherited, due to the origin of these enzymes in anaerobic organisms. Despite this, their overall structure and the nature of the active site cofactor have been finely tuned by evolution, resulting in impressively efficient catalysts with very high turnover rates that use exclusively Earth-abundant metals. It is interesting to note that hydrogenases display remarkable diversity in active site composition, which is the result of convergent evolution. Depending on the nature of metal ions housed within the active site, hydrogenases are classified into three distinct classes: [FeFe]-hydrogenases, [NiFe]-hydrogenases and [Fe]-hydrogenases (also called Hmd). Their peculiar features are briefly described below.

### 2.1. [FeFe]-Hydrogenases

[FeFe]-hydrogenases (FFHs) represent the most active group of hydrogenases, found in both prokaryotes and eukaryotes [26]. They are involved in hydrogen metabolism of several microorganisms, ranging from green algae to sulfate-reducing bacteria and many others. Putative FFH sequences have been identified in the genome of many organisms [27], and various classifications have been proposed [28]. A structural feature that is common to all FFHs is the so-called H-domain, a conserved ~40 kDa domain hosting the active site, where hydrogen chemistry takes place.

**Figure 1:**
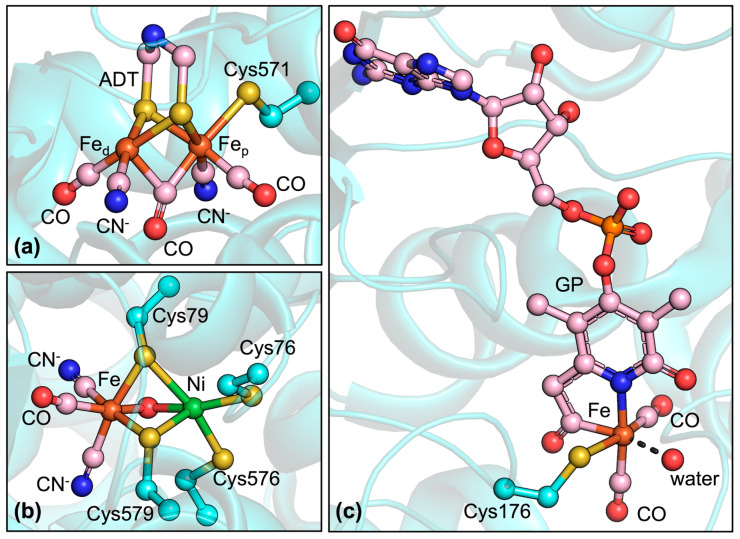
Structures of the catalytic cofactors found in hydrogenases. (**a**) [2Fe]H cluster of [FeFe]-hydrogenase from *Clostridium beijerinckii* (PDB code: 6TTL). ADT is the 2-aza-propane-(1,3)-dithiolate bridging ligand; Fe_d_ and Fe_p_ represent the distal and proximal Fe ions, respectively; the Cys571 sidechain is coordinated to both Fe_p_ and the [4Fe4S]H cubane cluster, which is not depicted in the figure. (**b**) NiFe dinuclear site of [NiFe]-hydrogenase from *Escherichia coli* (oxidized form, PDB code: 5A4M). (**c**) Mononuclear iron cofactor of [Fe]-hydrogenase from *Methanococcus aeolicus* (PDB code: 6HAC); GP represents the guanylylpyridinol ligand. Protein and protein-derived ligands are depicted in cyan; exogenous ligands are depicted in pink.

Catalytic activity requires a remarkably complex metal cofactor known as the H-cluster [29]. This metal cofactor is itself formed by two redox-coupled iron sites. One is an iron–sulfur cubane cluster, named [4Fe4S]H, similar to those found in some ferredoxins and other electron transfer proteins [30]. Other [4Fe4S] clusters are also present in FFHs and are probably involved in electron transfer. The other iron site, named [2Fe]H, is the cofactor where hydrogen evolution/splitting occurs. The [2Fe]H cluster (Figure 1a) is much more unusual in terms of both structure and iron coordination sphere composition, containing two iron ions connected by two bridging thiolate sulfur atoms coming from a 2-aza-propane-(1,3)-dithiolate (ADT) group and by a CO molecule. Additionally, another CO molecule and a CN^−^ anion are coordinated to each iron atom. One of the two irons (called proximal iron Fe_p_) is also bonded to a cysteinyl thiolate sulfur, which is shared with the [4Fe4S]H cofactor, making it 6-coordinated, while the other (distal iron or Fe_d_), maintains one vacancy to coordinate hydrogen.

Although some of them have been found to be more resilient, FFHs tend to be very O_2_-sensitive and are usually destroyed or inactivated even by trace amounts of oxygen [31,32] unless protecting conditions or reagents are employed [33,34]. The catalytic cycle of FFHs is quite complex and involves several intermediates, some of which have been extensively characterized by spectroscopic techniques; however, some aspects of the cycle remain to be clarified. The description of the catalytic cycle is beyond the scope of this review; thus, readers can refer to exhaustive papers [35,36] for a current overview of the proposed catalytic intermediates and their characterization.

### 2.2. [NiFe]-Hydrogenases

[NiFe]-hydrogenases (NFHs) are the most common type of hydrogenases [37], found in archaea, bacteria, and even eukaryotes. As the name suggests, their metal cofactor contains an iron and a nickel ion, but there are also other differences in metal coordination spheres compared to FFHs [38]. NFHs generally exhibit lower activity and reversibility compared to FFHs, and they are more commonly employed for hydrogen splitting rather than hydrogen evolution. Most NFHs characterized so far share a similar heterodimeric structure formed by a larger (named alpha, ~60 kDa) and a smaller (named beta, ~30 kDa) subunit. The larger subunit contains the active NiFe site, while the smaller subunit houses a variable number of [4Fe4S] clusters necessary for electron transfer to and from the NiFe site.

NFHs are commonly classified into four groups [39,40] depending on differences in active site composition, location in the cellular space, and function. There exists also a subgroup of NFHs with high O_2_ tolerance, known as [NiFeSe]-hydrogenases (NFSHs), in which one of the cysteine residues bonded to the nickel is replaced by a selenocysteine [41,42]. NFHs are usually more tolerant to oxygen than FFHs, being merely reversibly deactivated by oxygen, while some even retain their catalytic activity in air. Interestingly, O_2_-tolerant NFHs often contain a unique metal cofactor, a [4Fe-3S] 6-Cys cluster, in place of the proximal [4Fe-4S] 4-Cys cluster, which seems to be implicated in enzyme recovery [43,44]. As mentioned, the metal cofactor responsible for catalytic activity differs significantly from that of FFHs [45]. In NFHs, the nickel atom is coordinated by four cysteine thiolate ligands, two of which are also coordinated to the iron atom. The latter is also coordinated by a CO molecule and two CN^−^ anions, similarly to the FFH [2Fe]H cluster (Figure 1b).

The catalytic cycle of NFHs is still under investigation; however, thanks to much experimental effort, many of the main events and intermediates have been determined [46,47,48]. The first NFH reaction mechanism suggested heterolytic cleavage of the H–H bond [49]. Nevertheless, a possible homolytic cleavage mechanism cannot be excluded, as evidenced by subsequent computational investigations, which resulted in comparable energy barriers for the two mechanisms [50].

### 2.3. [Fe]-Hydrogenases

[Fe]-hydrogenases (FHs, also known as H_2_-forming methylenetetrahydromethanopterin dehydrogenases or Hmds), are significantly rarer than FFHs and NFHs, having been found only in methanogenic archaea, particularly when nickel availability is decreased [51]. They catalyze the stereospecific reversible hydride transfer between H_2_ and N^5^,N^10^-methenyltetrahydromethanopterin, the latter being a necessary cofactor for the process. This reaction is part of the hydrogenotrophic methanogenesis pathway in methanogens, which extracts reducing equivalents from hydrogen to reduce CO_2_ to methane gas. From a structural viewpoint, FH is a homodimeric protein formed by two 37 kDa subunits, joined by a shared alpha-helical bundle formed by the C-terminal regions, while the N-terminal region adopts a structure derived from the Rossman fold [52]. The metal cofactor present in FHs is unique and greatly different from those present in FFHs and FNHs, since it does not contain an iron sulfur cluster or other polynuclear clusters. Indeed, FHs were initially thought to be metal-free hydrogenases. However, it is now accepted that FHs contain a metal cofactor constituted by an iron ion bound through a pyridine nitrogen and an unusual acyl-iron bond to guanylylpyridinol (GP), a molecule constituting a guanine monophosphate unit bonded to pyridinol, in addition to two CO molecules and a Cys thiolate sulfur (Figure 1c). This cofactor is both heat- and light-sensitive, rapidly decomposing into guanylylpyridinol, CO, and free iron [53], which makes its manipulation and study somewhat cumbersome, even though the enzyme is O_2_-tolerant. A mechanism has been proposed for FHs on the basis of both experimental investigations [54,55] and computational studies [56,57].

## 3. Hydrogenase-Based Devices for Hydrogen and Energy Production

Scientific enquiry into the inner workings of hydrogenases is greatly motivated by the potentially useful application of these enzymes and of systems derived from them [58]. Since hydrogenases can catalyze both hydrogen splitting and hydrogen formation with high turnover and low overpotential, they are ideally suited for the construction of devices designed to produce hydrogen or exploit it as an energy source. To accomplish these tasks, hydrogenases should be incorporated or deposited on the electrodes of devices specifically designed to perform and electrically couple the required reactions [59]. The choice of electrode materials and incorporation of hydrogenases into the electrode are not trivial. However, several advances in this field have been recently accomplished by exploiting the properties of advanced materials [60,61,62,63,64]. NFSHs are usually preferred to other types of hydrogenases for practical applications, owing to their high activity combined with O_2_ tolerance.

The setup and detailed nature of electrochemical devices depends on the specific process desired. Two of the major types of devices, photoelectrochemical cells and biofuel cells, are discussed in further detail below. We are aware that it is impossible to exhaustively cover the huge amount of work in the field. Thus, several case studies are selected to focus on the most recent and promising results on the incorporation of hydrogenases into these kinds of electrochemical devices.

### 3.1. Photoelectrochemical Cells

**Figure 2:**
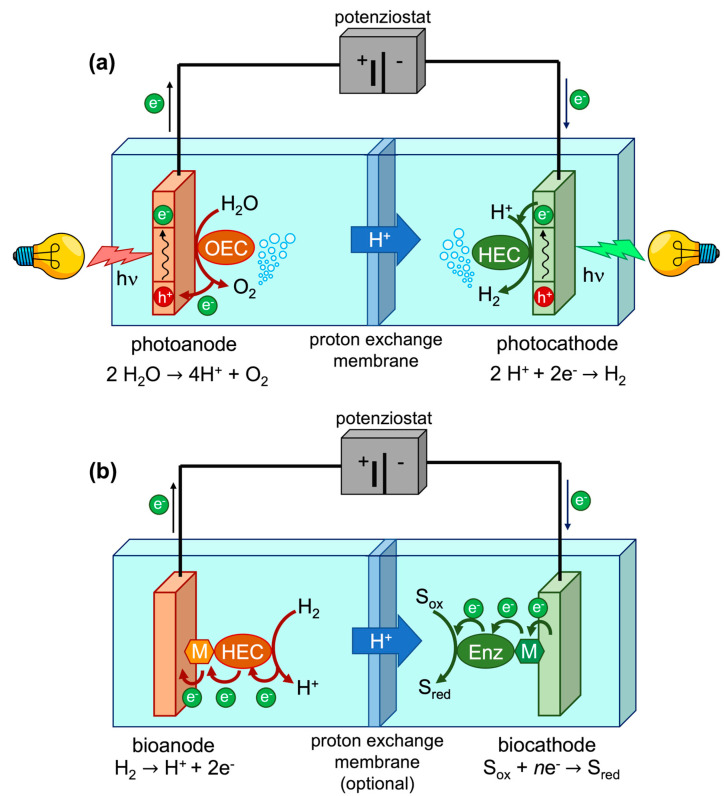
Schematic representation of (**a**) photoelectrochemical cell and (**b**) biofuel cell. OEC = oxygen-evolving catalyst; HEC = hydrogen-evolving catalyst. In panel (**a**), photoactive electrodes are exemplified as semiconductors. The red-circled h^+^ represents positively charged electron holes, which are generated upon light absorption. The green circled e− represents electrons. In panel (**b**), Enz represents a generic enzyme catalyzing the reduction of an oxidized substrate, S_ox_ to S_red_. M represents redox mediators interposed between the electrodes and the enzymes.

Hydrogen production can be performed by devices in which proton reduction to hydrogen, catalyzed by hydrogenase deposited onto the cathode, is electrochemically coupled to oxidation of another chemical species. When that species is water, the whole process produces H_2_ and O_2_ and is named water splitting. Water splitting can also be fueled by energy supplied as light [65], in which case it effectively represents a kind of artificial photosynthesis that can be performed through specialized devices named photoelectrochemical cells (PECs, Figure 2a). Notably, these devices require conjugation of catalytic activity to light absorption, which is commonly accomplished through the use of proper materials, such as semiconductors [66], or by functionalizing either the catalyst or the electrode surface with a suitable dye [67]. In such devices, an oxygen-evolving catalyst (OEC) is bound to the photoanode, while the photocathode is functionalized with a hydrogenase enzyme or, in general, a hydrogen-evolving catalyst (HEC).

Natural enzymes have been successfully immobilized on the surface of electrodes in various electrochemical devices, which are known as bio-photoelectrochemical cells (BPECs). These include a number of electrocatalytic biomolecules such as Photosystem I (PSI), Photosystem II (PSII) [68], bacterial photosynthetic reaction centers (RCs) [69], glucose oxidase (GO) [70], bilirubin oxidase (BO) [71], and laccase [72]. Even thylakoid membranes themselves have been incorporated in tailor-made devices [73]. Some recent and notable examples regarding the incorporation of hydrogenases in such devices are briefly described below.

Sokol et al. [74] proposed a remarkable design for a PEC based on Photosystem II (PSII) and a [NiFeSe]-hydrogenase that successfully accomplishes unassisted water splitting. In their device, the photoanode is formed by PSII wired to dye-sensitized titanium dioxide, while the cathode is wired to a [NiFeSe]-hydrogenase. The dye employed was a green light-absorbing diketopyrrolopyrrole, while electron transfer between the dye and PSII was enabled by a redox polymer, poly(1-vinylimidazole-*co*-allylamine)-Os(bipy)_2_Cl. In a previously designed similar PEC [75], in which light was absorbed only by PSII, a large external potential was needed to sustain the process, because of the low electrochemical potential of electrons produced by the PSII. This issue was resolved by employing the green light-absorbing dye.

TiO_2_ is one of the most commonly employed semiconductor materials for the electrodes in electrochemical devices and is also suited to the deposition of biocatalysts thanks to its porous surface, which is believed to facilitate the adsorption of proteins [76]. The efficiency of TiO_2_ light absorption can also be enhanced by manufacturing TiO_2_-based inverse opals (IO), materials with a highly organized internal arrangement giving rise to periodic modulation of the dielectric constant [77,78].

Nam et al. [79] developed a novel Si-based photocathode featuring a hierarchically structured inverse opal (IO)-TiO_2_ layer, optimized for integration of a [NiFeSe]-hydrogenase. The photocathode allowed stable and high hydrogenase load, and it was tested when coupled to both inorganic and biological photoanodes, based on BiVO_4_ and PSII, respectively, to perform the overall water-splitting process. Coupling to the BiVO_4_-based photocathode yielded a system capable of unassisted water splitting, while coupling to the PSII required application of an external voltage, albeit lower than reported by Sokol et al.

Perovskites have also been extensively explored as semiconductor materials for various applications including BPECs. These are described by the general formula ABX_3_, where A and B are cations of different size, and X is oxygen or a halide [80]. Perovskite-based materials have been rapidly improving in the last decade and have enabled the creation of photovoltaic cells with impressive power conversion efficiencies, now surpassing 29% [81].

In this context, Moore et al. [82] designed a sophisticated photocathode based on an organic–inorganic lead halide perovskite. Despite their high efficiency, these materials are sensitive to heat, air, and moisture; therefore, their employment is quite challenging. The authors were able to protect the perovskite layer by encapsulation, using a eutectic alloy, metal foil, and epoxy resin, reporting for the first time the integration of an NFSH with this kind of material. By using a BiVO_4_-based photoanode, they were also able to accomplish bias-free water splitting with higher solar-to-hydrogen efficiency than those illustrated above [82].

Lastly, Tian et al. [83] developed a PEC using ZnO-protected Cu_2_O, a material never used before for a hydrogenase photocathode. Cu_2_O presents favorable properties as a p-type semiconductor, albeit unstable. To increase its stability, the authors employed a protective ZnO layer, which also allows electron flow from the Cu_2_O layer. An FFH was then immobilized on the ZnO layer, and the electrode was integrated into a testing PEC device, confirming that, when illuminated, the photocathode evolved hydrogen. The protecting ZnO layer showed signs of degradation after prolonged operation, resulting in the formation of nano-sticks.

### 3.2. Biofuel Cells

Hydrogen oxidation to protons can be coupled to the reduction of other species in so-called fuel cells (FCs), which exploit the overall chemical reaction promoted by catalysts deposited onto the electrodes to generate electrical energy (Figure 2b) [84]. Conceptually, the counterpart of a PEC performing water splitting is an FC exploiting hydrogen oxidation to protons and oxygen reduction to water. When the catalysts used by the apparatus are enzymes, the device is known as a biofuel cell (BFC), in which hydrogenases are usually wired to the anode to catalyze the oxidation of hydrogen. A number of interesting hydrogenase-based BFC designs have been recently devised [58].

Gentil et al. [85] successfully immobilized an NFSH on carbon nanotube-modified electrodes using a rational design strategy. To this end, they grafted hydrophobic molecules to carbon nanotubes, such as anthraquinone and adamantane, selected to interact with the hydrogenase surface. Such modified nanotubes were able to bind the NFSH and were used to manufacture gas diffusion electrodes for a H_2_/air BFC operating at room temperature and pressure.

In another study, Hardt et al. [86] designed a redox-active film composed of an FFH embedded into a 2,2′-viologen-modified hydrogel to perform bidirectional and reversible hydrogen conversion. This film was used to manufacture a bioanode for a H_2_/O_2_ BFC achieving a 1.16 V open-circuit voltage. The same film was also usable as a highly efficient biocathode for hydrogen evolution. The authors proposed a detailed kinetic model to explain the observed catalytic activity.

Viologen-based polymers have been used extensively in BFC designs, and their properties can also be exploited to protect the sensitive hydrogenases from inactivation caused by oxygen, as proposed by Szczesny et al. [87]. In their recent work, the authors proposed a noteworthy design for a dual-gas breathing H_2_/air BFC, relying on a bilirubin oxidase-based biocathode and a NFSH-based bioanode. The bioanode is composed of two layers of a redox-active polymer which serves both as support and as protection for the NFSH from O_2_. The cell achieved an open-circuit voltage equal to 1.13 V. In a more recent study, the same authors were also able to design a H_2_/O_2_ BFC, comprising a hydrogenase/redox polymer-based bioanode integrating a highly active but O_2_-sensitive FFH, protected by the redox polymer [88].

An additional concern with hydrogenases is their thermal stability, which limits the temperature range in which BFCs can operate. To address this problem, Wang et al. [89] designed a novel bioanode using a thermostable and highly active NFH isolated in *Pyrococcus furiosus*. They first developed a thermophilic archaea host system to express the hydrogenase, which was then immobilized on a nanotube-modified carbon felt electrode. This bioanode was integrated in a H_2_/air BFC with a Pt/C cathode, successfully operating in the temperature range 40–80 °C with high efficiency.

The practical feasibility of biohybrid devices such as BPECs and BFCs has been clearly demonstrated by these technically impressive designs. Integration of natural hydrogenases and other biologically derived electrocatalysts with electrochemical devices has been accomplished using a variety of different strategies. Despite the incredible advances reported so far, a number of difficulties remain to be addressed. Many of the materials used to make these devices are delicate and quite sophisticated, inflating the cost of their wide adoption. The overall efficiency of the setup is also of paramount importance in this regard. Lastly, biohybrid devices also pose specific problems arising from the chemical fragility of biomolecules, which hamper long-term operation, as required for continuous energy/fuel generation. Solving these issues is a key aspect of the coming transition toward sustainable energy production, which will undoubtedly be the focus of much scientific inquiry in the foreseeable future.

## 4. Artificial Electrocatalysts for Hydrogen Production

Despite being conceptually very simple, the hydrogen evolution reaction (HER) is a deceivingly difficult process. The reaction is only kinetically viable when its overpotential is lowered by a suitable electrocatalyst (i.e., a hydrogen-evolving catalyst or HEC), serving to decrease the activation barriers involved in the various chemical steps. The best solid-state HEC is platinum [90], which displays several favorable properties, such as an almost-zero hydrogen adsorption free energy, durability, and high electrical conductivity. However, it is a rare and expensive material, and this makes its wide adoption unfeasible. Therefore, large-scale production of hydrogen, as required by a full shift toward renewable energy sources, is associated with the discovery of artificial electrocatalysts based on abundant transition metals such as Fe, Co, Ni, Cu, Mo, and W [91,92]. Numerous inorganic-based materials have been shown to be active as HECs, particularly sulfides, selenides, carbides, nitrides, and phosphides. In addition to heterogeneous catalysts, a variety of small-molecule metal complexes have been developed for homogeneous catalysis, aimed at reproducing the exceeding catalytic performances of hydrogenases into simple, robust, and easily tunable systems.

**Figure 3:**
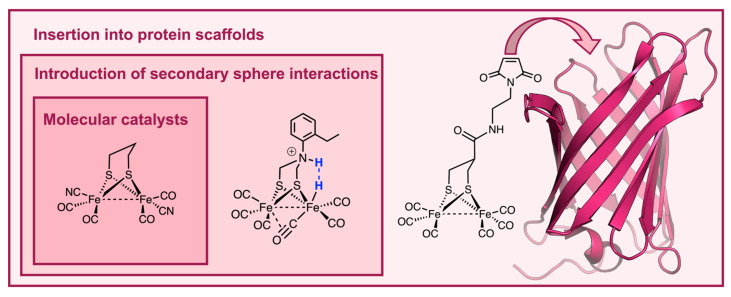
Modulation of redox and catalytic properties of hydrogenase molecular catalysts by introducing secondary coordination sphere interactions. Starting from the molecular complexes (**inner and darker box**), acid–base functionalities acting as proton shuttles are introduced (**middle box**). Then, the inclusion of catalytic centers into protein scaffolds provides artificial hydrogenases (**outer and lighter box**). Representative examples of diiron-containing catalysts are depicted: diiron propanedithiolate complex mimicking [FeFe]-hydrogenase in the primary coordination sphere [21]; N-aryl azadithiolate diiron hexacarbonyl complex [93]; maleimide-functionalized diiron propanedithiolate hexacarbonyl complex bound to nitrobindin (PDB code: 2A13) [94].

This section presents an overview of molecular catalysts conceived as electrocatalysts for HER. Starting from the smallest molecular scaffolds, organic and biomolecular architectures with increasing levels of complexity have been constructed around the catalytic metal site (Figure 3), introducing secondary sphere and long-range interactions that have a substantial impact on redox and catalytic properties. A summary of the catalytic parameters for electrochemical H_2_ production by selected catalysts is reported in Table 1.

### 4.1. Mimicking Hydrogenases with Small-Molecule Complexes

Inspired by hydrogenases, a plethora of molecular complexes have been reported as structural and functional mimics of the active sites of these enzymes. Studies on these mimics have afforded further insight into the behavior and properties of their natural counterparts, as thoroughly described in the literature [21,95,96,97,98,99]. Indeed, deep investigation of some hydrogenase mimics enabled the elucidation of a number of details regarding the catalytic mechanism through isotope experiments, as described in a recent study [100]. The majority of model complexes are inspired by the diiron dithiolate carbonyl cluster of [FeFe]-hydrogenases, and well-established synthetic strategies are currently available [101,102,103]. The first generation of such structural mimics dates back to 1999, when limited structural information on hydrogenases active sites was available. Complexes bearing azadithiolate (ADT) and oxadithiolate ligands were reported in [104,105], until the nature of the bridgehead atom in the propanedithiolate (pdt) moiety was identified as nitrogen. Since then, substantial work has been focused on preparing model compounds resembling natural [FeFe]-hydrogenases in terms of primary coordination sphere and oxidation state of the diiron center [21]. However, most of these synthetic complexes usually catalyzed proton reduction at high overpotential. Strategies to overcome this major drawback involved either substitution of the carbonyl ligands or modification of the ADT bridge [106,107,108,109]. Abiotic phosphine or carbene ligands [110,111] are found in compounds with redox properties that are the closest to the thermodynamic potential for proton reduction. These ligands mimic the electronic properties of cyanide and increase the electron density of the diiron center, thus favoring protonation [112]. Another effective method for modulating the redox properties of diiron model systems is the inclusion of a properly substituted aromatic dithiolate bridgehead [113,114]. In particular, the use of a benezenedithiolate (bdt) ligand bearing electron-withdrawing substituents allowed lowering the overpotential for catalysis but compromised the catalytic performances in terms of turnover frequency.

**Figure 4:**
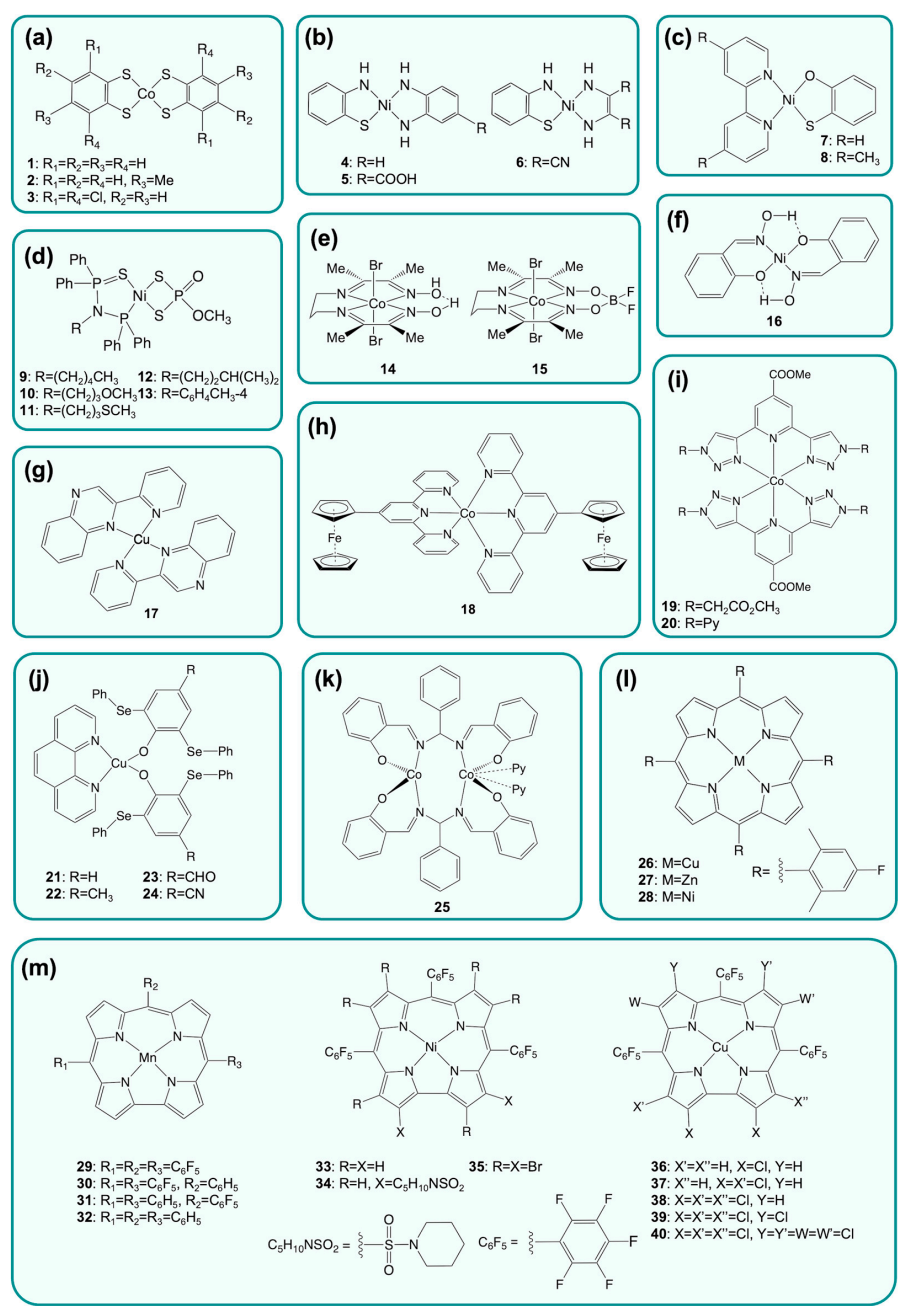
Significant examples of small-molecule classes of HECs discussed within the review. In some cases, more than one complex is shown for each class. (**a**) Cobalt dithiolene complexes [117]; (**b**) heteroleptic thiolate diamine nickel complexes [122]; (**c**) heteroleptic diamine oxathiolate nickel complexes [123]; (**d**) aminodiphosphine monosulfide nickel complexes [124]; (**e**) cobalt diimine dioxime complexes [128]; (**f**) nickel salicylketoxime complexes [129]; (**g**) copper quinoxaline complexes [155]; (**h**) cobalt polypyridine complexes [131]; (**i**) bistriazolylpyridine cobalt complexes [132]; (**j**) selenoether phenolate copper complexes [133]; (**k**) binuclear cobalt complexes based on the *N*,*N*′-bis(salicylidene)-phenylmethanediamine ligand [134]; (**l**) metalloporphyrins [135]; (**m**) metallocorroles [136,137,138].

In addition to hydrogenase active site mimics, a variety of molecular HECs have been developed through a carefully designed electronic environment provided by the interaction between metal and ligands [115] (Figure 4). Examples of this are complexes with non-innocent S-donating ligands [116,117], such as dithiolene, thiosemicarbazone, and ene-1,2-dithiolate, which display some unique properties due to their unusual electronic structure (Figure 4a). Both experimental and computational studies revealed that the presence of redox-active ligands in these complexes is strongly linked to the catalytic activity [118]. Recently, Niu et al. [119] synthesized new dithiolene complexes of nickel able to evolve hydrogen in both aqueous and nonaqueous media. The compound with the highest performance ([BzPy]_2_[Ni(tdas)_2_]; BzPy = benzyl pyridinium, tdas = 1,2,5-thiadiazole-3,4-dithiolate) reached a turnover frequency (TOF) of 5.36 min^−1^ at a potential equal to −0.99 V (vs. SHE) in acetonitrile solutions (Table 1, entry 1). The same compound showed a higher TOF of 9.25 min^−1^ at −0.49 V (vs. SHE) in neutral buffer solutions (Table 1, entry 2). The electronic structure of the metal complex can also be rationally modified by imposing structural constraints on ligand geometry, as proposed by Drosou et al. [120]. Alkylation of two dithiolene sulfur atoms with a propyl moiety afforded compounds ([Ni(pbdt)]; pbdt = 2,2′-(propane-1,3-diylbis(sulfanediyl))dibenzene thiol) with increased metal basicity, able to reduce protons from TFA in DMF (Table 1, entry 3), albeit at comparable or higher overpotentials than their non-alkylated counterpart. Novel compounds based on bis(1,2,5-thiadiazole-3,4-dithiolate) ligands were prepared by Yin et al. [121] which were active as HECs in both DMF (Table 1, entry 4) and neutral buffer (Table 1, entry 5).

Kamatsos et al. [122] investigated three heteroleptic compounds bearing different diamine ligands and 2-aminobenzenethiolate (2-amnt, Figure 4b and Table 1, entry 6), active as electrocatalysts for proton reduction in DMF with TFA as a proton donor. Albeit reaching remarkably high TOF (6120 min^−1^), the highest catalytic current for these complexes was observed at very high overpotentials. Replacement of nitrogen with an oxygen atom in the 2-amnt ligand yielded analogous heteroleptic diamine oxathiolate nickel complexes (Figure 4c and Table 1, entry 7). This enabled catalysis at lower overpotentials, but significantly decreased both TON and TOF [123]. In another recent study, Chen et al. [124] synthetized five novel nickel complexes bearing o-methyldithiophosphate and aminodiphosphine monosulfide ligands (Figure 4d), which showed HEC activity in acetonitrile using TFA as a proton source. The electrocatalytic studies on these complexes showed that the catalytic performances depend on the nature of the substituent in the aminodiphosphine monosulfide ligand. In particular, the presence of a 4-CH_3_C_6_H_4_ group leads to the highest TOF and the lowest overpotential (Table 1, entry 8) due to the electron-donating effect.

Complexes including nitrogen and oxygen-based ligands have also been extensively studied as HECs, and several classes have emerged as promising, including diamine dioxime, polypyridine, corrole, porphyrin, tetraimine, diglyoxime, and cyclam complexes, in most cases containing cobalt [125]. Cobalt diimine dioxime compounds (Figure 4e) have long being known as HECs [126] and several water-soluble analogues have also been reported. In particular, Peters and coworkers evaluated the effect of the bridging groups within the macrocycle and found that the proton bridged compound ([Co(DO)(DOH)pn(OH_2_)_2_]^2+^, Table 1, entry 9) displayed the highest TON (23) among the series, catalyzing proton reduction at notably low overpotential (260 mV) [127]. A recent study by Sun et al. [128] granted further insight into the mechanism. The authors synthesized and studied two cobalt diimine dioxime complexes and carried out a detailed quantitative analysis of their catalytic activity, which confirmed a previously proposed catalytic mechanism.

**Table 1:** Catalytic parameters of artificial H_2_ evolving electrocatalysts discussed in the review.

Entry	Catalyst	Solvent (Proton Source)	pH	TON	TOF ^[a]^ (min^−1^)	η ^[b]^ (mV)	Duration (h)	Ref.
1	[BzPy]_2_[Ni(tdas)_2_]	MeCN (AcOH)		1286	5.36	520	4	[119]
2	[BzPy]_2_[Ni(tdas)_2_]	H_2_O	5	2220	9.25	520	4	[119]
3	[Ni(pbdt)]	DMF (TFA)				760		[120]
4	[Bz-1-MeIm]_2_[Ni(tdas)_2_]	DMF (AcOH)		70.3	0.58	941.6	2	[121]
5	[Bz-1-MeIm]_2_[Ni(tdas)_2_]	H_2_O	7	1546	12.9	837.6	2	[121]
6	[Ni(2-amnt)(dmnt)]	H_2_O/DMF (TFA)			6120	1040		[122]
7	[Ni(bpy)(mp)]	DMF (Bu_4_N^+^PF_6_^−^)		39.2	786	720	3	[123]
8	[Ni((PPh_2_)({S}PPh_2_) C_6_H_4_CH_3_(S_2_P{O}OCH_3_)]	MeCN (TFA)			4.24 × 10^4^	610	2	[124]
9	[Co(DO)(DOH)pn(OH_2_)_2_]^2+^	H_2_O	2.2	23		260	24	[127]
10	Ni-SAO	H_2_O	7	1428	23.8	837.6	1	[129]
11	[Co(ppq)(MeCN)(TfO)]^+^	DMF (Et_3_NH^+^BF_4_^−^)			3.36 × 10^5^	600		[130]
12	[Co(Fc-tpy)_2_]^2+^	DMF/H_2_O 95/5 (AcOH)			4.95 × 10^4^	655	2	[131]
13	[Co(ptim)_2_]^2+^	MeCN (AcOH)			0.99	370	12	[132]
14	[Co(mtim)_2_]^2+^	MeCN (AcOH)			0.24	300	12	[132]
15	Cu(phen)(4-CHO-2,6-SePh-PhO)_2_	DMF (AcOH)			19.5	610		[133]
16	[Co_2_(spmd)_2_Py_2_]^+^	DMF (AcOH)			26.7	460	5	[134]
17	CuTFP	MeCN (BA)			1518			[135]
18	[Ni(pfsc)]^−^	MeCN (TFA)			61.7	360		[136]
19	F15C-Mn	DMF (TsOH)			1.42	1400		[137]
20	F15C-Mn	H_2_O/MeCN 3/2	5.5		25.9	1027	8	[137]
21	Cu-Cl8	MeCN (TFA)			1.47 × 10^7^	360	6	[138]
22	Ni(P^Ph^_2_N^EtOMe′^_2_)_2_]^2+^	MeCN (4-CNAnH^+^BF_4_^−^)		8	30	140		[139]
23	[Co(P^Ph^_2_N^Ph^_2_)(CH_3_CN)_3_]^2+^	MeCN (Bu_4_N^+^BF_4_^−^)			5400	285		[140]
24	(μ-S_2_(CH_2_)_2_N(2-ethylphenyl)[Fe(CO)_3_]_2_ ^[c]^	H_2_O	5.5	1.0 × 10^6^	2406	180	2.5	[141]
25	Co-corrole-crown-ether	PC/H_2_O (BA)			5.93 × 10^5^			[142]
26	[Fe_2_(bdt)(CO)_6_]	H_2_O/SDS	3	52	1.56 × 10^5^	500	1	[143]
27	[Fe_4_(Zn-L)_6_] ^[d]^	MeCN (Et_3_NH^+^BF_4_^−^)			5.04 × 10^7^	640		[144]
28	[[Ni(P^Ph^_2_N^DPE^_2_)_2_]^2+^	MeCN/H_2_O (DMFH^+^OTf^−^)			8.4 × 10^4^	330		[145]
29	Co-MP11	H_2_O	7	2.5 × 10^4^	402	852	0.4	[146]
30	*Ht*-CoM61A	H_2_O	7	2.7 × 10^5^		830	6	[147]
31	Co-MC6*a	H_2_O	6.5	2.3 × 10^5^		680	1.4	[148]
32	SwMb-[Co(dmgH)_2_(H_2_O)_2_] ^[e]^	H_2_O	7	3.2		200		[149]
33	HO-[Co(dmgH)_2_(H_2_O)_2_]	H_2_O	7	6.3		460		[150]
34	SA-[Co(BioPy_2_tacn)(MeCN)]^2+^	H_2_O	3.5			780	3	[151]
35	NiRd ^[f]^	H_2_O	4.5		4800	540		[152]
36	V08D-NiRd	H_2_O	4.0		3.0 × 10^5^	600		[153]
37	Ni(II)-NBP	H_2_O	5	210	1	560	1	[154]

^[a]^ TOF is the maximum TOF observed in the best experimental conditions. ^[b]^ η is the overpotential where the maximum catalytic current is observed. ^[c]^ Catalyst physiabsorbed on edge plane graphite electrode. ^[d]^ TOF was extrapolated at [H^+^] = 1 M based on TOF_max_ = 2*k_cat_[H+]^0^. ^[e]^ Catalyst absorbed on C100 multiwalled carbon nanotubes (MWCNTs). ^[f]^ Experiments performed at T = 4 °C.

A nickel salicylketoxime complex (Figure 4f) was instead reported by Wang et al. [129] to be active as an electrocatalyst in neutral aqueous buffer, achieving a TON of 1428 over 1 h of electrolysis (Table 1, entry 10). Additionally, the first Cu(I) quinoxaline complex (Figure 4g) active as an HEC was reported by Drosou et al. [155], and its activity was investigated by electrochemical and DFT methods, which also provided insight into the reaction mechanism. Polypyridine complexes (Figure 4h) have also been abundantly explored as potential HECs, as summarized in some excellent reviews [156,157]. Novel 5- and 6-coordinated cobalt complexes with polypyridyl ligands were designed by Liu et al. [130], and their properties were studied both experimentally and theoretically. Both complexes were found to be active HECs when using triethylammonium tetrafluoroborate (Et_3_NH^+^BF_4_^−^) as the proton source in DMF, with the most active complex ([Co(ppq)(MeCN)(TfO)]^+^; Table 1, entry 11) reaching a remarkably high TOF of 336,000 min^−1^. Voltammetry experiments also allowed demonstrating the dissociation of a phosphine axial ligand from one of the two complexes, prior to hydrogen evolution. An unusual cobalt complex was proposed by Kumar Padhi et al. [131], in which terpyridine molecules were conjugated to ferrocene ([Co(Fc-tpy)_2_]^2+^; Table 1, entry 12). The activity of the complex was tested in DMF/H_2_O solutions with acetic acid as the proton source and compared to the parent terpyridine compound lacking the ferrocene moieties. The complex conjugated to ferrocene showed a 200 mV lowered overpotential and a doubled TOF compared to the simple terpyridine cobalt derivative. These results were attributed to the electron-donating character of ferrocene. Another interesting example of sophisticated ligands was provided by Cui et al. [132], who prepared two bistriazolylpyridine cobalt complexes (Figure 4i and Table 1, entries 13–14) through click chemistry and performed a number of spectroelectrochemical experiments on them, also involving the use of cobaltocene as reductant. Both resulted active HECs in acetonitrile with acetic acid, and their redox and catalytic behaviors were shown to be influenced by substitution on the triazole moiety. The synthesis of copper(II) phenolate selenoether complexes (Figure 4j) was recently accomplished by Upadhyay et al. [133], who showed that these compounds were air-stable and catalytically active as electrocatalysts. The presence of electron-withdrawing groups on the ligands (e.g., formyl group) was also demonstrated to yield the highest TOF values (Table 1, entry 15). Lastly, some binuclear cobalt complexes (Figure 4k and Table 1, entry 16), recently reported by To et al. [134], were also found to be highly active HECs in DMF with acetic acid as the proton source.

As mentioned, numerous porphyrin-based complexes have also been employed as homogeneous HECs (Figure 4l) [158,159,160,161]. The different mechanisms possible for hydrogen evolution by porphyrins were compared and discussed in a review on the subject [162]. Chou et al. [135] recently prepared TFP (5,10,15,20-tetrakis(4-fluoro-2,6-dimethylphenyl)porphyrin) complexes of Cu(II), Zn(II), and Ni(II). Among them, only the Cu(II) derivative exhibited proton reduction activity in acetonitrile with benzoic acid as the proton source (Table 1, entry 17). Novel corrole complexes of Co, Cu, Mo, Mn, and Ni have also been described in very recent work (Figure 4m) [136,137,138,163]. Gross and coworkers reported a nickel corrole derivative displaying HER activity in acetonitrile with low overpotential (360 mV, Table 1, entry 18) [136]. A manganese corrole catalyst was shown to catalyze proton reduction in both DMF and mixed aqueous/acetonitrile solution (Table 1, entries 19–20), reaching the highest TOF in the latter condition, but suffering from a high overpotential (1.027 V) [137]. To the best of our knowledge, the most active corrole-based catalysts are the copper complexes developed by Sudhakar and Panda [138], who evaluated the effect of chlorine substitution on electrocatalytic hydrogen evolution. The octafluoro-corrole derivative (CuCl8, Table 1, entry 21) showed an impressively high TOF (1.47 × 10^7^ min^−1^) for proton reduction in acetonitrile with low overpotential (360 mV).

Overall, an impressive amount of research effort has been directed toward the development of small-molecule complexes mimicking hydrogenase function. Despite the achievement of notable results, there is still ample margin for improvement, particularly regarding the overpotentials, TOF, costs, and toxicity of the proposed compounds.

The catalytic activity and overpotentials of molecular HECs depend on a number of factors, including the identity of the metal and its ligands, electronic structure of the complex, and operational conditions. Minimization of the overpotential down to a nearly vanishing value is a crucial aspect to obtain synthetic analogues of natural hydrogenases. However, optimizing the electronic structure and substitution of metal complexes to achieve minimum overpotential is a challenging task, since hard ligands favor protonation of a low-valent metal atom, but hinder proton reduction [164]. Moreover, non-innocent ligands might lower the overpotential by allowing storage of reducing equivalents, as proposed for dithiolene complexes [116]. The presence of internal bases has also been shown to decrease the overpotential by facilitating protonation/deprotonation steps in the catalytic cycle [21]. Due to this intricate interplay of factors, it is difficult to delineate a unique structure–function relationship for all the hydrogen-evolving catalysts, although correlations between substitution and catalytic activity have been reported for selected systems [165].

### 4.2. Building Complexity around the Catalytic Center

Small-molecule complexes offer the advantage of being easily produced through synthesis. Although active as HECs, the lack of the protein environment hampers them in reproducing the efficiency of the natural counterparts, such as the capability of performing well in water. Indeed, the unique ability of hydrogenases to reversibly catalyze proton reduction is certainly connected to their highly evolved protein structures [166]. Despite the apparent simplicity of HER, this reaction involves electron transfer (ET) and proton transfer (PT) steps, which are usually interconnected as proton-coupled electron transfer (PCET) processes [167,168]. Incorporating acid–base groups into molecular electrocatalysts facilitates this mechanism by positioning the proton donor and acceptor close to each other, thus resembling natural catalysts.

Following nature’s footpath, the insertion of proton relays near the active site of synthetic complexes has been actively pursued. Several mononuclear nickel *bis* diphosphine catalysts, incorporating a pendant amine group in the ligand backbone ([Ni(P^R’^_2_N^R^_2_)_2_]^2+^ complexes), have been developed to investigate the role of the base in catalysis [169,170]. Interestingly, the catalytic activity for complexes containing properly positioned pendant amines occurs at much higher rates and/or lower overpotentials, compared to analogous catalysts lacking the base group (Table 1, entry 22) [139]. Such improvement has been attributed not only to a facilitated proton transfer from the solution to the catalytic site, but also to a decrease in the activation barrier for H–H bond formation (or cleavage) [139,171,172,173]. Substitution of Ni(II) with Co(II) afforded complexes able to catalyze electrochemical H_2_ production only when a pendant amine was incorporated in the diphosphine ligand, further confirming the essential role of the proton relay in the catalytic reaction (Table 1, entry 23) [140]. This approach was fruitfully applied also to structural mimics of the [FeFe]-hydrogenase active site. Dey and coworkers recently developed a series of azadithiolate (ADT) diiron hexacarbonyl complexes carrying an *ortho*-substituted aryl moiety bound to the bridging nitrogen of ADT [141]. *Ortho*-substitution of the aryl group favors a tetrahedral geometry at the nitrogen atom, which disfavors electron delocalization to the aromatic ring, in contrast with the nearly planar geometry observed in other N-aryl ADT complexes. This leads to an increased electron density at the well-oriented, pyramidal nitrogen, enabling its protonation at pH 5.5, whereas unsubstituted N-aryl derivatives show proton reduction activity only in strongly acidic conditions (Table 1, entry 24). Thus, despite not affecting the formal redox potential of the diiron center, the presence of an active proton shuttle allows HER to occur at very low overpotential (180 mV). In follow-up work, such systems were shown to promote both proton reduction and H_2_ oxidation as natural enzymes, and they were implemented in the construction of a fuel cell [93]. Along these lines, Rauchfuss and coworkers developed, for the first time, a synthetic model containing the three functional components of the [FeFe]-hydrogenase active site: the reactive diiron center, an amine as the proton shuttle, and a ferrocene derivative as a one-electron redox partner [174].

Other relevant examples of this strategy are the “hangman porphyrins”. These complexes typically contain a xanthene ring that places a pendant carboxylate or amine group over the metal ion [175,176,177]. The boosting effect of introducing an acid–base functionality was first demonstrated by Nocera and coworkers, who reported the lower overpotentials and improved catalytic activities of hangman porphyrins compared to the non-hangman analogues. These systems also provided insights into the mechanism of HER catalyzed by cobalt, nickel, or iron porphyrins, mainly based on electrochemical and spectroscopic data coupled to DFT analysis [162,178,179]. Starting from this pioneering work by Nocera and coworkers, the hangman strategy has been subsequently expanded, by appending increasingly complex molecular frameworks to porphyrin-like catalysts [180,181,182]. As a notable example, Cao and coworkers designed a hydrogen-bonded water network by positioning a crown ether over a cobalt corrole catalyst, which significantly enhanced the catalytic performances by facilitated proton delivery (Table 1, entry 25) [142]. However, hangman porphyrins, as the majority of molecular complexes discussed above, have been typically studied in organic solvents due to their scarce solubility in water. This represents an important drawback for their potential application in fuel cells.

Several alternative strategies have been proposed for the construction of secondary sphere interactions around the metal-containing catalysts, concurrently enabling good performance in water. For example, these strategies foresee the inclusion of molecular hydrogenase catalysts into cyclodextrins [183,184], micelles [143], and metal–organic supramolecular cages [144,185]. Encapsulation of a sulfonated derivative of ADT diiron hexacarbonyl into β-cyclodextrin provided water solubility to the insoluble complex, but disfavored electrochemical proton reduction, as demonstrated by increased overpotentials. This phenomenon was ascribed to the steric constraints imposed by the cyclodextrin cavity, hampering structural rearrangements during the catalytic cycle or inhibiting proton delivery to the active site [183,184]. Conversely, dispersion of benzenedithiolate diiron hexacarbonyl into micelles formed in aqueous sodium dodecyl sulfate (SDS) provided efficient electrochemical H_2_ evolution (Table 1, entry 26), occurring at an overpotential of <500 mV (pH 3) [143], which is competitive with the values reported for most molecular catalysts. The encapsulation of a pyridine-substituted diiron hydrogenase model into a synthetic Zn-porphyrin cage (Fe_4_(Zn-L)_6_) significantly decreased the catalytic overpotential (by 150 mV) at the expense of a slightly lower catalytic rate with respect to the same complex in freely diffusing conditions (Table 1, entry 27) [144].

### 4.3. Engineering Hydrogenase Activity into Protein Scaffolds

As described above, with the aim of tuning and controlling the activity of artificial H_2_ evolving electrocatalysts, more and more sophisticated and diverse environments have been continuously exploited. As a step further in the development of artificial hydrogenases closely resembling the natural counterparts, the field has broadened to include more elaborate systems based on the construction of a peptide- or protein-based architecture to host the catalytic center. Small peptides binding hydrogenase model complexes lay in the middle ground between natural metalloenzymes and small-molecule complexes, and they provide the chance to carefully modulate secondary sphere interactions while also allowing activity in water. Early work in this field was focused on anchoring dithiolate bridged diiron hexacarbonyl clusters to peptide scaffolds, mimicking the active site of [FeFe]-hydrogenases. Dutton and coworkers reported the first peptide-based structural model of hydrogenases consisting of a de novo designed helical peptide capable of binding the diiron cluster through cysteine residues [186]. Along these lines, other groups have developed [FeFe]-hydrogenase models endowed with redox activity, exploiting either natural or artificial dithiol containing peptides to bind the diiron core [187,188,189]. Notably, Jones and coworkers reported an alternative strategy to link a dithiolate bridged diiron core to a peptide scaffold, which does not involve thiolates as anchoring sites. In this approach, an unnatural phosphine amino acid was incorporated into the peptide sequence, for binding the diiron cluster and replacing a terminal carbonyl [190]. The resulting phosphine-bound diiron pentacarbonyl complex displayed improved electrocatalytic properties, which were ascribed to the higher electron donating properties of the phosphine compared to CO, acting as a mimic of CN^−^ found in hydrogenases [191]. Shaw and coworkers introduced an amino-acid-based outer coordination sphere by further modification of the diphosphine ligand with a mono- or dipeptide in the previously described [Ni(P^R’^_2_N^R^_2_)_2_]^2+^ complexes [145,192,193]. In particular, simple incorporation of a diglycine ethyl esther (DPE = dipeptide esther) into the ligand framework imparted water solubility to this class of molecular catalysts while boosting their catalytic activity both in proton reduction and in H_2_ oxidation (Table 1, entry 28) [145]. The carboxylic groups of glycines may facilitate proton delivery from the solvent to the metal and vice versa, creating a minimal proton channel [192].

Despite countless mimetics of the [FeFe]-hydrogenase diiron cluster being reported, functional models of [NiFe]-hydrogenases are rare [194,195]. The absent or little activity displayed by structural mimics of these enzymes underlines that the protein environment plays a fundamental role in dictating the properties of the metal cluster. Dutta et al. reported a synthetic methodology to construct minimal peptide-based model of [NiFe]-hydrogenases using a fragment derived from the enzyme nickel superoxide dismutase (NiSOD) [196]. Although the synthetic approach is promising, functional peptide-based models of this class of enzymes are still missing.

**Figure 5:**
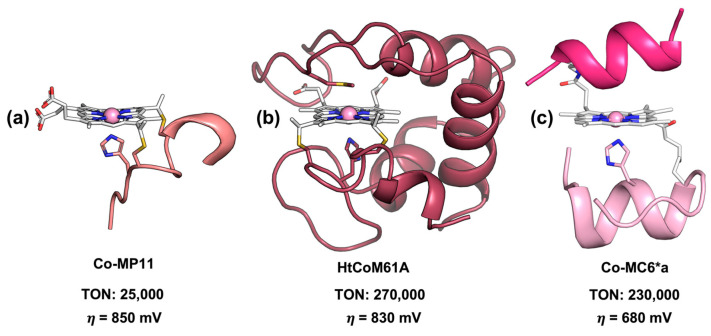
Peptide/protein co-porphyrin conjugates: (**a**) Co-MP11 [146] (PDB code: 1CRC); (**b**) HtCoM61A [147] (PDB code: 1AYG); (**c**) Co-MC6*a [148] (designed model). TON and overpotential (η) of electrocatalytic hydrogen evolution are reported for each catalyst.

The effect that a protein matrix can exert on catalyst robustness and overpotential was demonstrated by Bren and coworkers, who screened cobalt porphyrins covalently bound to peptide scaffolds with different levels of complexity (Figure 5). Among them, the first and simplest was Co-MP11, which consisted of the cobalt-substituted cytochrome c-derived heme-peptide complex, known as microperoxidase-11 (Figure 5a and Table 1, entry 29). Co-MP11 performed ~25,000 turnovers in electrocatalytic hydrogen evolution at an overpotential of ~850 mV, but showed a significant drop in the activity after only 15 min of electrolysis [146]. Conversely, the cobalt-substituted mutant of Hydrogenobacter thermophilus cytochrome c552 (Ht-CoM61A, Figure 5b, Table 1, entry 30) was more robust, lasting for 6 h of electrolysis and displaying a 10-fold enhanced turnover number (~270,000 TON). However, almost no improvement in the catalytic overpotential of Ht-CoM61A (830 mV) was observed with respect to Co-MP11 [147]. This drawback was recently addressed thanks to the joint efforts of the Bren and Lombardi labs, with the synthetic cobalt deuteroporphyrin-containing miniprotein Co-MC6*a (Figure 5c and Table 1, entry 31) [148,197]. Co-MC6*a promotes proton reduction from nearly neutral aqueous solutions (pH 6.5) with the highest catalytic current at an overpotential of 680 mV, which is a significantly lower value compared to both Co-MP11 and Ht-CoM61A. Furthermore, investigations of Co-MC6*a revealed that enhancing peptide folding via the addition of TFE (2,2,2-trfifluoroethanol) as a cosolvent lowered overpotential to 520 mV, while increasing catalyst longevity up to 1.3 h with respect to Co-MP11 [148]. Remarkably, this miniature enzyme performed more than 230,000 turnovers, a value comparable to Ht-CoM61A.

Artificial hydrogenases have been also produced by incorporating small-molecule catalysts into protein scaffolds, with the aim of imparting enzyme-like features to synthetic complexes. This approach, usually referred to as the host–guest strategy [198,199,200], takes advantage of stable and evolutionary selected protein architectures to create a tunable protein environment around a catalytic metal site. Cobalt bis-glyoxime catalysts have been incorporated into several natural proteins replacing the native metal cofactor. Artero and coworkers reported two biohybrid hydrogenase catalysts via reconstitution of apo sperm-whale myoglobin (SwMb) with two different cobaloxime moieties (Table 1, entry 32) [149]. The electrocatalytic activity of the cobalt centers was retained in both systems, which performed proton reduction in aqueous conditions with low overpotential (~200 mV). However, the operational stability of these biohybrids remained limited to few turnovers (TON = 3.2 at pH 7). Slight improvements in terms of TON (up to 12) were observed upon insertion of the same cobaloxime catalysts into the heme-binding pocket of heme oxygenase (HO) as the host protein (Table 1, entry 33) [150]. In both cases, the complexes alone showed better performance compared to when embedded into the proteins. This finding was ascribed to a bimolecular mechanism involving two molecules, which is inhibited when the catalysts are housed within the protein scaffold. Ghirlanda and Fillol developed cobalt-based biohybrid systems exploiting the biotin–streptavidin technology, which relies on the exceedingly high binding affinity of the protein streptavidin (SA) toward biotin [151]. Biotinylated aminopyridine cobalt complexes encapsulated into SA retained their electrocatalytic activity, displaying a decreased overpotential compared to the lone complexes (Table 1, entry 34).

The simple substitution of iron with nickel into *Desulfovibrio desulfuricans* rubredoxin led to an efficient hydrogenase model (NiRd), showing an electrocatalytic behavior closely resembling that of *Desulfovibrio vulgaris* [NiFe] hydrogenase, with H_2_ production rates that are comparable to those of the native enzyme (Table 1, entry 35) [152]. A library of NiRd mutants was generated using site-directed mutagenesis to evaluate the effect of mutations in the secondary coordination sphere. Interestingly, the introduction of a carboxylate residue, mimicking that found in the [NiFe] hydrogenase, considerably increased the turnover frequency, likely resulting from a proton transfer pathway into the active site, without significantly affecting the overpotential (Table 1, entry 36) [153]. Recently, a rational metalloprotein design approach was applied by Chakraborty and coworkers to repurpose a copper storage protein (Csp1) into a nickel-binding protein (NBP) able to promote hydrogen evolution [154]. A tetrathiolate nickel-binding site was engineered into Csp1, inspired by the active site of [NiFe] hydrogenases. Ni-NBP performed 210 turnovers over 1 h of bulk electrolysis, displaying an overpotential which is still far from those of native hydrogenases, but comparable to those of most molecular catalysts (Table 1, entry 37).

## 5. Bioinspired Systems for Light-Driven Hydrogen Production

Photocatalytic hydrogen evolution is the most attractive process, as, in principle, it could exploit sunlight as the energy source. At the same time, it is also the most challenging, since it requires the presence of three components working in synergy: a photosensitizer, a catalyst, and an electron donor [201]. A suitable light-harvesting unit should be characterized by a strong absorption and a long-lived excited state to facilitate charge separation. Furthermore, the redox potentials of the photosensitizer and catalyst should be well matched, allowing the desired charge transfer among them to occur. Lastly, as H_2_ evolution is a reductive process, an electron reservoir is required to bring the photosensitizer back to the initial state. Sacrificial electron donors are typically used to this purpose, but coupling proton reduction with an oxidative process (such as water oxidation in photosynthesis) is highly desirable. Several reagents have been used as sacrificial electron donors in photocatalytic hydrogen evolution, with the most used being ascorbic acid (AscOH), alcohols, and aliphatic amines [202,203]. The choice of the electron donor is as important as that of the photosensitizer, since it must be oxidized to an inert species which does not interfere with catalysis. However, the use of a sacrificial reagent represents a limitation to the scale-up of the process, because it requires large quantities or repeated addition of the reagent, which may not be affordable from a sustainability and/or an economic point of view. One possibility to address this problem consists of using electron donors which can be reversibly regenerated upon light irradiation, as reported by Girault and coworkers [204,205].

In a homogeneous catalytic system, the energy transfer is limited by diffusion, which brings the active components close enough for the process to occur. Possible strategies to optimize the efficiency of the photoelectron transfer consist of creating covalent links between the catalyst and the photosensitizer or of gathering the active components into a micro- or nanometer-sized reactor. This problem is not encountered with heterogenous catalysts, as light harvesting and catalysis occur in the frame of a photoactive material. Since 1970s, there has been growing interest in the fabrication of highly efficient semiconductor-based photocatalysts, which include TiO_2_ [206], ZnO [207], Fe_2_O_3_ [208], and CdS [209]. Heterogeneous systems offer the advantage of being highly stable and long-lived; however, unlike molecular catalysts, they have limited light-harvesting abilities and tunability. Further, these materials suffer from other common drawbacks, including wide bandgaps, conduction band potentials lower than the proton reduction potential, toxicity, and high cost. The need to overcome the inherent limitations of inorganic materials has prompted great interest towards the study of organic polymers as photocatalysts [210]. The first and possibly the most studied photocatalytic polymeric semiconductor is graphitic carbon nitride (g-C_3_N_4_), due to its facile synthesis, appealing electronic band structure, and high physicochemical stability [211]. In the past few years, a new class of highly porous organic polymers emerged as prominent candidates for photocatalytic hydrogen evolution, namely, covalent organic frameworks (COFs) [212,213,214]. COFs are made up by molecular building blocks; hence, they grant the possibility of modulating the different functions, fundamental to the photocatalytic process: light harvesting, charge separation, charge transport, and catalysis. Furthermore, COFs are characterized by nanometer-sized structural pores entailing a high interaction surface area and providing accessibility to sensitizers, sacrificial components, and cocatalysts throughout the material [215]. A comprehensive analysis of heterogeneous systems for hydrogen production is beyond the scope of this review; interested readers can refer to the literature [216,217]. In the remainder of this section, homogeneous photocatalytic systems involving molecular and biomolecular components are described. A summary of the catalytic parameters from selected photocatalytic H_2_ production systems, described below, is reported in Table 2.

### 5.1. Light-Harvesting Units in Photocatalytic Hydrogen Production

**Figure 6:**
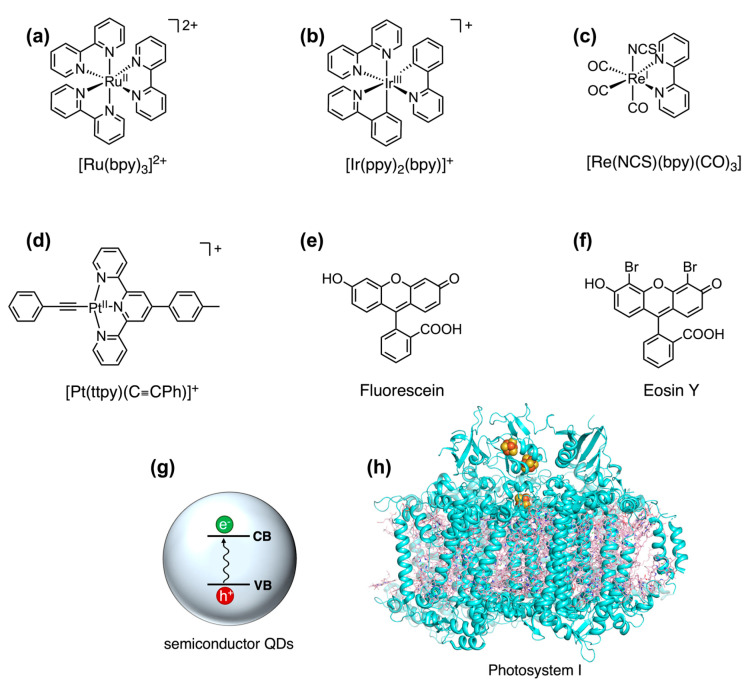
Representative examples of photosensitizers employed in the photocatalytic systems discussed in this review: (**a**) [Ru(bpy)_3_]^2+^; (**b**) [Ir(ppy)_2_(bpy)]^+^ [218]; (**c**) [Re(NCS)(bpy)(CO)_3_] [226]; (**d**) fluorescein; (**e**) eosin Y; (**f**) [Pt(ttpy)(C≡CPh)]^+^ [227]; (**g**) semiconductor QDs; (**h**) photosystem I (PDB code: 1JB0).

The choice of the most appropriate photosensitizer is of paramount importance in light-driven catalysis. Ruthenium, iridium, rhenium, and platinum polypyridine complexes are usually preferred (Figure 6a–d), since they display intense metal-to-ligand charge transfer absorption bands in the visible region, populating a long-lived excited-state, which provides enough time for the electron transfer [246,247]. However, their high cost, toxicity, and difficult recyclability hamper their use on a large scale, thus prompting research toward noble-metal free photosensitizers. In contrast, organic dyes such as fluorescein and eosin Y (Figure 6e,f) show higher absorption intensity of light and fluorescence quantum efficiency compared to metal complexes, but have generally lower chemical stability and shorter excited-state lifetime [248,249].

In order to overcome these issues, the use of semiconductor quantum dots (QDs) as photosensitizers has gained much interest (Figure 6g) [250,251]. QDs are nanocrystals with a diameter of few nanometers, characterized by a unique electronic structure which is intermediate between that of bulk semiconductors and discrete atoms. Due to their small dimensions, the charge carriers (electron and holes) experience a quantum confinement effect; thus, they can occupy only discrete energy levels. As a result, QDs display strong absorption and fluorescence at specific wavelengths. [252,253]. These wavelengths can be precisely tuned by altering the QD composition and size. In particular, the energy gap between the conduction and valence bands increases as the size of QDs decreases, providing extended stabilization to the charge-separated state [254]. The highest photoluminescence quantum yields have been reported for cadmium containing nanocrystals, such as CdS, CdSe, and CdTe; thus, research on environmentally friendly and highly efficient QDs is growing [255,256].

Nature developed an astonishing light-harvesting unit, namely, photosystem I (PSI, Figure 6h). It is the light-harvesting machinery found in bacterial reaction centers and works in synergy with photosystem II (PSII) to achieve photosynthesis [201,257]. PSI is provided with a molecular antenna made of multiple carotenoid and chlorophyll molecules on the protein periphery. Light excitation initiates a cascade of rapid electron-transfer steps between the cofactors, starting with the primary electron donor (P700) and terminating with a [4Fe-4S] cluster (F_B_). The quantum yield of PSI approaches 1; thus, almost every absorbed photon is converted to the charge-separated state (P700^+^ F_B_^−^), which is characterized by a long lifetime (~60 ms) [258]. The impressive photosensitizing properties of PSI have attracted much interest in switching its photocatalytic activity from NADH synthesis to hydrogen evolution, taking advantage of its ability to provide electrons to partners other than natural ones [259]. Biohybrid systems have been constructed by combining molecular or biomolecular catalysts with PSI.

### 5.2. Photo-Activated Catalysts for Hydrogen Production

#### 5.2.1. Molecular Catalysts

Over the last few decades, great efforts have been focused on the design of photocatalytic systems coupling a [FeFe]-hydrogenase model complex with a variety of light-harvesting units [218,260,261,262,263]. Unlike native [FeFe]-hydrogenases, their mimics undergo gradual degradation and loss of CO under light irradiation over a few hours, resulting in relatively low TON [22]. Substitution of one carbonyl with a phosphine ligand results in improved photostability, but also increases the electron density at the diiron site [264,265]. This effect, which is beneficial for electrocatalysis, is detrimental for the photoinduced process, because it disfavors the charge transfer from the photosensitizer. The use of weakly donating phosphines such as P(pyr)_3_ (tris(N-pyrrolyl)phosphine) has been exploited for the construction of functional photocatalysts, performing up to 466 turnovers over 8 h of irradiation upon matching with a iridium(III) photosensitizer (Table 2, entry 1) [218]. Other strategies aimed at increasing the stability of diiron carbonyl complexes consist of replacing the aliphatic dithiolate bridged ligand with an aromatic one, such as benezenedithiolate (bdt) [266]. Due to their structural rigidity, aromatic bridgeheads enhance catalyst robustness and offer the possibility of easily tuning redox properties by introducing substituents on the conjugated system [125]. The incorporation of electron-withdrawing substituents such as chlorine atoms on the aromatic bridging ligands was proven to facilitate the electron transfer from the photosensitizer, but compromised complex stability by weakening the Fe–S bonds (Table 2, entry 2) [114,219]. This problem was addressed by Hou and coworkers, who exploited a dinaphthalene-derived dithiolate ligand (μ-BNT) for the construction of an efficient photo-activated catalyst [220]. The photocatalytic system composed of the dinaphthalene diiron complex, eosin Y as a light-harvester, and triethylamine (TEA) as a sacrificial reagent was capable of performing 404 turnovers under 4 h of visible-light irradiation in a mixed water/acetonitrile solution (Table 2, entry 3), reporting the highest TON among the aromatic bridged diiron carbonyl complexes. Another subset of [FeFe]-hydrogenase mimics was developed by incorporating hydrophilic groups in the dithiolate bridge, increasing water solubility of the complexes. In this context, Wu and coworkers developed two diiron model complex bearing sulfonate-substituted thiol ligands, using either a bridged dithiolate (Fe_2_S_2_-SO_3_Na) or two separate thiolate ligands (Fe_2_S_2_–2SO_3_Na) [221]. The two analogues displayed similar catalytic behavior, which was found to be strongly dependent on the nature of the photosensitizer. In detail, TON values of 114 and 178 were reported in the presence of [Ru(bpy)_3_]^2+^ (Table 2, entries 4–5), while values of 18,800 and 26,500 were found in the presence of CdSe QDs (Table 2, entries 6–7) for the bridged and unbridged complex, respectively. This comparative study confirmed the importance of an efficient electron transfer, from the photosensitizer to the catalytic unit, in photocatalytic hydrogen-evolving systems, highlighting the superior performances of QDs with respect to ruthenium(II) polypyridyl complexes [221].

**Figure 7:**
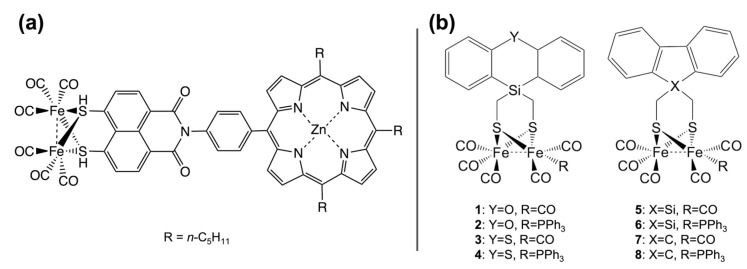
Molecular dyads consisting of [FeFe]-hydrogenase model complex covalently linked to a photosensitizer unit. (**a**) ZnP−NMI-Fe_2_S_2_(CO)_6_ (ZnP = 5-phenyl-10,15,20-tri-n-pentylporphyrin; NMI = naphtalene monoimide) [269]; (**b**) [FeFe] complexes tethered to fluorene and silafluorene derivatives [222].

One possible strategy to enhance the stability and performances of a homogeneous photocatalytic system consists of constructing supramolecular assemblies, where the distance between the catalyst and the light-harvesting unit can be precisely controlled. Covalently linked dyads were constructed associating diiron carbonyl complexes with several kinds of photosensitizers, where the organic dithiolate bridge serves as a connection between the components [267,268,269,270,271] (Figure 7). Rigid and conjugated organic molecular structures are preferred as spacers between the catalytic and the photosensitizer units, in order to favor the charge transfer process. However, moderate activities have been reported for covalent dyads containing zinc porphyrins [268,269] (Figure 7a), rhenium complexes, or ruthenium complexes as photosensitizers. A new class of organic, silicon-containing photosensitizer was recently reported, displaying the advantage of fast electron transfer through the Si–CH_2_–S linker [222,272] (Figure 7b). The system composed of 1-silafluorene linked to diiron dithiolate hexacarbonyl complex performed 539 turnovers over 7 h of light irradiation under optimized reaction conditions (Table 2, entry 8) [222].

In addition to model complexes of hydrogenases, a variety of synthetic molecular catalysts have been studied for photocatalytic hydrogen production. Among them, Ni-based catalysts have demonstrated excellent catalytic activity and received much attention among researchers. A series of mononuclear nickel(II) complexes with diphosphine ligands, developed for electrochemical proton reduction, were also tested for light-driven catalysis. Remarkably, the [Ni(P^R’^N^R^P^R’^)_2_]^2+^ complex, reported by DuBois and coworkers, was tested by Holland and Eisenberg et al. in the presence of different photosensitizers, displaying long-term stability and performing 2700 turnovers when paired to [Ru(bpy)_3_]^2+^ (Table 2, entry 9) [223]. In addition to the phosphine ligand, complexes with dithiolate or aminothiolate ligands were also developed, taking inspiration from the ligand composition of [NiFe]-hydrogenase active sites. Pyridinethiolate complexes such as [Ni(X-pyS)_3_]^−^ (pyS = pyridine-2-thiolate; X = 5-H, 5-Cl, 5-CF_3_, 6-CH_3_) and [Ni(4,4′-Y-2,2′-bpy)(pyS)_2_] (Y = H, CH_3_, OCH_3_) displayed impressive photocatalytic properties and performed up to 7300 turnovers in the presence of fluorescein as photosensitizer (Table 2, entry 10) [224]. Interestingly, in the absence of substituents on the pyS ligand, the nickel complex assumed a square planar geometry, while distorted octahedral geometries were found in the other cases. Mechanistic studies showed that a key step in the catalytic cycle of these catalysts is the reversible dechelation and protonation of a pyridine nitrogen, which enables the formation and coupling of a metal hydride with the N-bound proton to produce H_2_ [224]. On the basis of these findings, nickel(II) bischelate complexes with a square-planar coordination have more recently gained attention [123]. The [Ni(bpy)(mp)] (mp = 2-hydroxythiophenol) complex displayed notable durability, being capable of promoting H_2_ evolution under light irradiation for more than 100 h, reaching exceedingly high turnover numbers (Table 2, entry 11).

Cobalt-based catalysts have been also privileged candidates for hydrogen production, due to their relatively low cost and high tolerance to O_2_ [125,149,273]. A wide variety of cobalt complexes have been tested for light-driven hydrogen evolution, mostly involving macrocyclic or pseudomacrocyclic square-planar ligand arrays. Among them, difluoroboryl-bridged cobaloximes such as [Co(dmgBF_2_)_2_] (dmg^2−^ = dimethylglyoximato) have been the most studied, owing to their stability against hydrolysis in acidic conditions [274]. Encouraging results were obtained by Artero and coworkers, which explored the use of cyclometallated iridium diamine ([Ir(ppy)_2_(phen)]^+^; ppy = 2-phenylpyridine, phen = 1,10-phenantroline) or tricarbonylrhenium diamine ([ReBr(CO)_3_(phen)]) as photosensitizers achieving 165 and 273 TON, respectively (Table 2, entries 12–13) [225]. H_2_ production was improved by enhancing the long-term stability of the rhenium photosensitizer upon substitution of the labile bromide ligand with thiocyanate [226]. With the highly stable photosensitizer [Re(NCS)(bpy)(CO)_3_], the stability of cobaloxime catalyst became performance-limiting, and up to 6000 turnovers with respect to the rhenium complex were performed (Table 2, entry 14). Eisenberg et al. also focused on photocatalytic activity of cobaloxime complexes, with a platinum terpyridyl acetylide ([Pt(ttpy)(C≡CPh)]^+^, ttpy = 4′-p-tolylterpyridine) [227] or eosin Y as photosensitizers [275], achieving up to 2400 TONs with the Pt complex (Table 2, entry 15). Cobalt porphyrins have been predominantly studied for electrochemical proton reduction, and only limited reports are available for photochemical H_2_ evolution with this class of catalysts (Table 2, entries 16–17) [228,229]. A notable example was reported by Hung et al., who developed a sulfonate-substituted tetraphenyl porphyrin (CoTPPS) capable of performing ~6400 turnovers in neutral water and in the presence of [Ru(bpy)_3_]^2+^ as photosensitizer [229].

#### 5.2.2. Peptide- and Protein-Based Catalytic Systems

Combining hydrogenase model complexes with peptides or protein scaffolds has proven to be a valuable strategy to modulate the surrounding environment of the catalyst, providing additional stabilization and/or altering the charge transfer mechanism. Feng and coworkers constructed protein-based nanoreactors by incorporation of [FeFe]-hydrogenase mimics into protein-based supramolecular structures [230,231]. First, the authors achieved catalyst encapsulation into horse spleen apoferritin ([FeFe]-HSF) [230]. The negatively charged inner surface of apo-HSF, due to the presence of multiple glutamate residues, was exploited to cage up to 312 diiron carbonyl moieties. The photocatalytic hydrogen evolution activity was studied in mild acidic aqueous solutions, using [Ru(bpy)_3_]^2+^ as photosensitizer and ascorbate as sacrificial electron donor (Table 2, entry 18). Interestingly, even though penetration of the ruthenium complex into the protein interior was negligible, photocatalytic activity per single diiron catalyst was improved ~8-fold upon inclusion into apo-HSF. This finding suggests that the electron transfer process does not need the catalyst and the photosensitizer to be in close proximity, but it may occur through a multistep electron tunneling pathway across the protein matrix. Similar results were reported in a subsequent work, where the diiron dithiolate complex was entrapped into self-assembling protein nanogels formed by ovalbumin (Table 2, entry 19) [231]. Remarkably, the composite nanogel ([FeFe]-OVA) showed a 15-fold improvement in photocatalytic activity compared to the freely diffusing catalyst, suggesting an enhanced electron transfer efficiency mediated by the protein framework.

Artificial hydrogenases obtained by protein design and engineering have also been studied for light-driven catalysis. Hayashi and coworkers anchored the diiron carbonyl cluster to cytochrome c, exploiting the cysteine residues in the CXXC heme-binding motif [232]. Substitution of the native cofactor with the synthetic diiron complex endowed the resulting conjugate ([FeFe]-Cyt c) with photocatalytic H_2_ production activity in the presence of [Ru(bpy)_3_]^2+^ as a photosensitizer and ascorbate as a sacrificial electron donor (Table 2, entry 20) [232]. In a follow-up study, aimed at improving the efficiency of electron transfer, the authors selected an 18-residue fragment (Pep18) of the cytochrome c sequence as a peptide matrix to covalently bind both the diiron carbonyl core and the photoactive ruthenium complex (Table 2, entry 21) [189]. In particular, the native heme coordinating histidine was chosen as the anchoring site for [Ru(bpy)(tpy)]^2+^ (bpy = 2,2′-bipyridine, tpy = 2,2′:6′,2″-terpyridine), and the two cysteines in the CXXCH sequence acted as ligands for the diiron complex. The intramolecular photochemical system ([FeFe][Ru]-Pep18) promoted H_2_ evolution in aqueous solution at pH 8.5 in the presence of excess ascorbate as sacrificial reagent, albeit displaying a limited turnover number (TON ~9). Addition of the imidazole-bound Ru complex ([Ru(bpy)(tpy)(im)]^2+^) as an external photosensitizer to the [FeFe]-Pep18 conjugate, did not lead to H_2_ evolution, evidencing that efficient electron transfer might occur only within the peptide framework. Further expanding the range of protein scaffolds, the Hayashi group covalently attached the diiron cluster into a rigid β-barrel protein, i.e., nitrobindin (NB), containing a suitable cavity to host the metal complex [94]. Inclusion of the [FeFe]-hydrogenase active site mimic within the barrel yielded an artificial metalloenzyme ([FeFe]-NB) capable of performing up to 130 turnovers over 6 h of activity in the presence of [Ru(bpy)_3_]^2+^ as photosensitizer (Table 2, entry 22). The catalytic complex without the protein matrix displayed similar total hydrogen production but required only 2 h to reach the plateau. The decreased initial reaction rate was attributed to a lowered accessibility of the ruthenium complex to the diiron site when embedded in the protein matrix.

**Figure 8:**
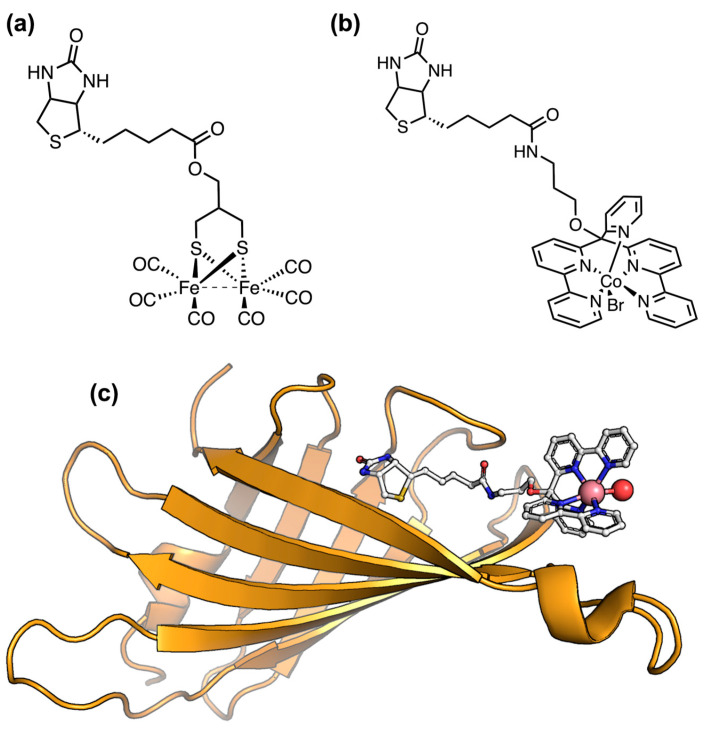
Biotinylated photocatalytic HECs anchored to SA through the biotin–SA technology. (**a**) Diiron hexacarbonyl complex [233]; (**b**) cobalt pentapyridine ([CoBr(appy)-Biot]) complex [234]. Panel (**c**) shows the crystal structure of the [CoBr(appy)-Biot]-SA biohybrid (PDB code: 6FRY).

Biomimetic diiron hexacarbonyl complexes have also been encapsulated into SA using the biotin–SA technology (Figure 8a and Table 2, entry 23) [233]. Incorporation in the protein scaffold provided the diiron catalyst ([FeFe]biot-SA) with prolonged stability and enhanced activity compared to the isolated complex.

The conjugation of synthetic complexes to proteins has not been limited to structural mimics of hydrogenases; it has also been applied with other classes of molecular catalysts. Ward and coworkers incorporated a biotinylated cobalt pentapyridyl complex ([CoBr(appy)-Biot]) into several SA mutants (Figure 8b,c and Table 2, entries 24–25) to evaluate the effect of the amino acids close to the catalytic center on H_2_ evolution [234]. The presence of lysine residues near the metal site resulted not only in increased turnover number with respect to the wildtype protein, but also in a higher reaction rate, suggesting a beneficial proton shuttling effect.

Ghirlanda and coworkers inserted cobalt protoporphyrin IX (CoPPIX) into myoglobin, replacing the native heme cofactor. When coupled with Ru(bpy)_3_^2+^ in the presence of ascorbate, the beneficial role of the protein scaffold was demonstrated by a threefold increased TON (518 at pH 7) of Co-Mb with respect to the free porphyrin (Table 2, entry 26) [235]. In a subsequent work, the same authors explored the effect of replacing heme with CoPPIX in Cytochrome b_562_ [236]. Cofactor substitution afforded a photoactive enzyme (Co-cyt b_562_), whose activity could be modulated through mutagenesis in the primary coordination sphere (Table 2, entries 27–29). In particular, substitution of the axial methionine with an alanine or an aspartate residue led to a ~2.5-fold improvement in total hydrogen production with respect to the wildtype protein. Such enhancement was attributed to a synergic effect of the increased basicity at the metal center, which shifted the redox potential toward more negative values and facilitated proton transfer, with the aspartate acting as a proton relay.

Along these lines, Mahy and coworkers recently developed an artificial hydrogenase via insertion of maleimide functionalized CoPPIX into an artificial protein, named αRep (Table 2, entry 30) [237]. This protein has been shown to adopt a closed shell-like conformation hosting a large hydrophobic cavity, which has been previously exploited for the anchoring of catalytic metal complexes. Theoretical studies suggested that covalent anchoring of CoPPIX to αRep resulted in the complete encapsulation of the cofactor into the protein interior, with a glutamine sidechain acting as an axial ligand to the cobalt ion. The Co-αRep biohybrid showed hydrogen evolution photoinduced activity in the presence of [Ru(bpy)_3_]^2+^ as the photosensitizer and ascorbate as the electron donor, performing up to 163 TONs over 25 h of irradiation. As observed by Ghirlanda and coworkers, incorporation of CoPPIX into the protein scaffold significantly enhanced catalytic performance as compared to the lone porphyrin in the same experimental conditions. A substantial contribution to this field was granted by Bren and coworkers, who showcased the extraordinary catalytic potential of peptide–cobalt–porphyrin conjugates in light-driven hydrogen production [238,239]. On the basis of the promising results obtained in electrochemical catalysis, both Co-MP11 and Co-MC6*a were screened for photocatalytic activity in the presence of [Ru(bpy)_3_]^2+^ as a photosensitizer. In the photochemical system, Co-MP11 showed extended longevity, performing up to 2390 turnovers over 20 h of catalysis (Table 2, entry 31) [238]. This finding is in contrast with electrochemical studies, where substantial loss of activity was found after only 15 min of electrolysis. The short lifetime of Co-MP11 in electrocatalysis was attributed to porphyrin degradation by direct interaction with the electrode surface. Remarkably, Co-MC6*a outperformed Co-MP11 in terms of both catalyst durability (40 h) and turnover number (10,400), placing among the most efficient photocatalysts reported so far (Table 2, entry 32) [239]. In terms of catalyst longevity, Co-porphyrin-based artificial hydrogenases can be ordered as follows: Co-MC6*a > Co-αRep > Co-MP11 > Co-cyt b_562_ ≈ Co-Mb > CoP > CoTPPS. It can be noted that all the biohybrid systems display higher longevity with respect to the molecular metalloporphyrins CoP and CoTPPS, possibly resulting from a protective effect exerted by the protein matrix against light-induced catalyst degradation. This effect is much more evident when Co-porphyrin is covalently linked to the protein, as observed for Co-MP11, Co-αRep, and Co-MC6*a. Additionally, the higher durability of Co-MP11 compared to catalysts possessing a proper protein scaffold was attributed to tight histidine coordination in the covalently bound peptide [238]. Lastly, Co-MC6*a showed the highest longevity among Co-porphyrin biohybrids, despite its reduced dimensions. This was granted by the distal peptide chain which is designed to strongly interact with the porphyrin, thus acting as a shield against catalyst degradation [276].

**Figure 9:**
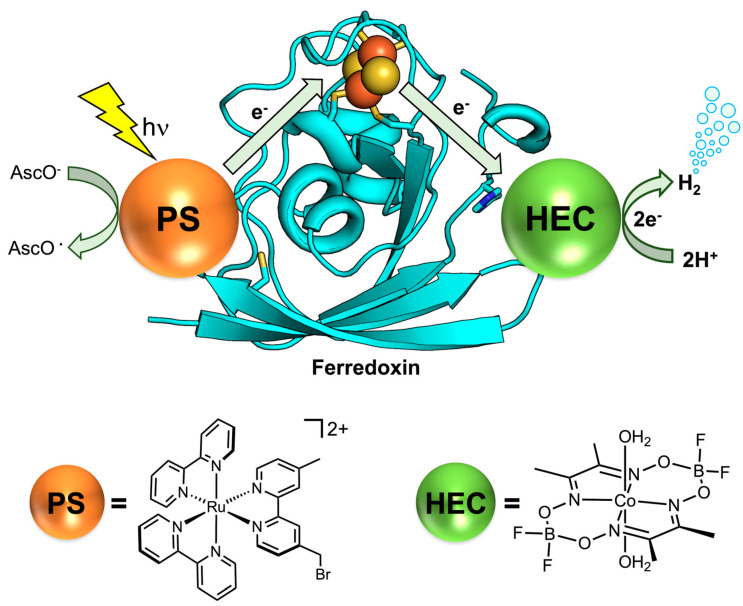
Biohybrid photocatalytic system composed of a ruthenium photosensitizer (PS = [Ru(4-CH_2_Br-4′-CH_3_-2,2′-bpy)(bpy)_2_]^2+^) and a cobaloxime catalyst (HEC = Co(dmgBF_2_)_2_·2H_2_O) linked to *Spinacia oleracea ferredoxin* (PDB code: 1A70) [242]. The iron and sulfur atoms of 2Fe2S center of ferredoxin are represented as spheres. The sidechains of residues involved in the binding of metal complexes are depicted as sticks. AscO^−^ and AscO^•^ represent the reduced and oxidized forms of ascorbate, respectively.

An alternative approach for constructing nature-inspired photocatalytic systems has involved the use of the photosynthetic protein PSI in place of molecular photosensitizers [258]. Utschig and coworkers applied this strategy to develop biohybrid systems by combining cobalt or nickel containing molecular catalysts with PSI. Self-assembly of the cobaloxime catalyst Co(dmgH)_2_pyCl (dmgH = dimethylglyoximato, py = pyridine) with PSI provided the biohybrid system containing 2–4 cobalt catalyst units per PSI (Table 2, entry 33) [240]. This cobaloxime–PSI hybrid was capable of promoting hydrogen evolution with one of the highest rates (TOF = 170 min^−^^1^) reported for artificial photocatalysts, performing up to 5200 turnovers. However, significant loss of catalytic activity was observed after relatively short times (1.5 h), which was ascribed to cobaloxime dissociation from the protein. Subsequently, the DuBois nickel diphosphine complex was assembled with PSI either via direct interaction or delivery by apo flavodoxin (apoFld, Table 2, entries 34–35) [241]. The [Ni(P_2_^Ph^N_2_^Ph^)_2_]-PSI biohybrid displayed a lower turnover frequency (44 min^−1^) and turnover number (1870) compared to the cobaloxime–PSI biohybrid. However, when the association to PSI was mediated by apoFld, the biohybrid reached 2865 turnovers in aqueous solution at nearly neutral pH, a value that is comparable to that displayed by the parent nickel molecular complex in acetonitrile in strongly acidic conditions [172]. Despite PSI-based biohybrids rapidly producing hydrogen, the large size of PSI (350 kDa) and the presence of multiple spectroscopically overlapping [4Fe-4S] cofactors hamper a thorough spectroscopic characterization and monitoring of the catalytic events. In subsequent work, the same authors designed a covalently assembled three component system by linking a cobaloxime catalyst and a ruthenium photosensitizer to *Spinacia oleracea ferredoxin* (Fd, Figure 9 and Table 2, entry 36) [242]. The small (10.5 kDa) electron transfer protein served as a scaffold to anchor the catalyst and the photosensitizer while also facilitating the charge transfer between them. Whereas the Ru–Fd–Co biohybrid performed up to 320 turnovers, no significant activity was observed when native Fd was replaced with apo-Fd, suggesting that the photoinduced electron transfer proceeds through the [2Fe2S] cofactor.

**Figure 10:**
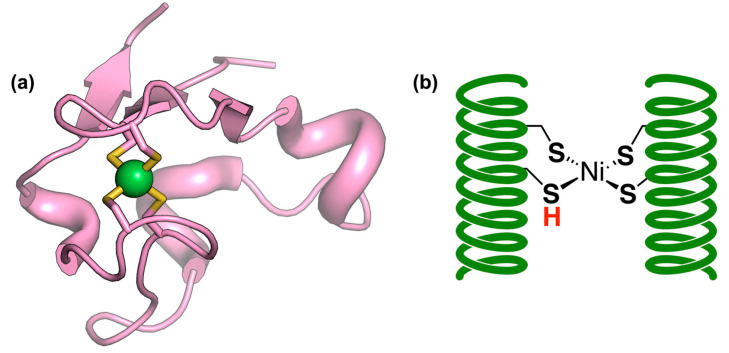
Engineered and de novo designed nickel-binding catalysts for photocatalytic hydrogen production. (**a**) X-ray crystal structure of nickel substituted rubredoxin (PDB code: 6NW0) [153]; (**b**) schematic representation of the nickel binding de novo designed 2SCC reported in [244]. One of the cysteine thiols is protonated to highlight its role in catalysis.

Shaafat and coworkers designed an even smaller biohybrid system by covalently assembling a ruthenium photosensitizer ([Ru^II^(2,2′-bipyridine)2(5,6-epoxy-5,6-dihydro- [1,10]-phenanthroline)]^2+^) to the previously described nickel-substituted rubredoxin (NiRd, Figure 10a and Table 2, entry 37) [243]. Attachment of the ruthenium complex to NiRd was provided through a native free cysteine residue, close to the metal-binding site. The effective distance between the nickel center and the photosensitizer was modulated by developing a series of mutants, featuring a free cysteine at different positions in the sequence. All the RuNiRd variants were capable of producing hydrogen upon light-irradiation, displaying enhanced activity with respect to the bimolecular Ru-complex/NiRd system. Furthermore, the activity of the biohybrid constructs was strongly dependent on the Ru–Ni distance calculated on the basis of rubredoxin crystal structure, suggesting an intramolecular electron-transfer pathway.

Inspired by the nickel center of [NiFe]-hydrogenases, Chackraborty and coworkers recently developed artificial metalloenzymes by engineering a tetrathiolate nickel center (NiS_4_) into de novo designed coiled coils [244,245]. In their first study, self-assembly of two de novo peptides, each bearing a CXXC motif, in the presence of nickel(II) yielded a two-stranded coiled coil (2SCC) enclosing the NiS_4_ site (Figure 10b and Table 2, entry 38) [244]. The artificial enzyme was able to produce H_2_ under photocatalytic conditions with a bell-shaped pH dependence, reaching its maximum activity at pH 5.6. Spectroscopic studied revealed that a precise acidity is required for activity, suggesting that the protonation state of Cys is crucial for H_2_ production. In the latest study, a peptide sequence was designed to favor a four-stranded coiled coil (4SCC), and the presence of a rubredoxin-like CXXC motif in each peptide strand was conceived to provide a dual nickel-binding site (Table 2, entry 39) [245]. Interestingly, nickel binding induced an oligomerization shift, stabilizing the formation of dimers, while the apo form was predominantly trimeric. This behavior indicates that nickel induced dissociation of the assembly by imposing its coordination preferences, which were stabilized in the 2SCC, as found in previous work. Notably, the newly designed Ni-4SCC peptide displayed a slightly improved activity compared to the 2SCC analogue. Even though these de novo metalloenzymes display relatively low turnover numbers, they represent a significant expansion of the de novo design approach for the development of artificial metalloproteins able to catalyze energy-relevant processes.

## 6. Conclusions and Future Perspectives

In the last few decades, research interest toward hydrogen production has greatly exploded, prompted by the urgency to face the current energy crisis. Hydrogen has a huge potential as a clean fuel, due to its incredibly high energy density and the possibility to be combusted without generating CO_2_. However, the development of cost-effective and environmentally friendly methods for hydrogen production is far from trivial, and much effort has been devoted to the elaboration of enzyme-based devices. These typically involve the use of hydrogenases either for light-driven hydrogen production (photoelectrochemical cells) or its conversion into electricity, upon matching of hydrogen oxidation with a reductive chemical reaction (biofuel cells). Despite hydrogenases showing impressive catalytic properties, their application in large-scale processes is currently hampered by difficulties with their expression and their limited tolerance to ambient oxygen. Extensive research has been conducted to elucidate the catalytic mechanism of hydrogenases, with the aim of replicating their function into artificial and tailorable systems. Since the late 1990s, a plethora of molecular catalysts have been proposed as hydrogenase mimics. While some have focused on reproducing the structure of the [FeFe]-hydrogenase active site, a wide variety of HECs have also been developed, combining redox non-innocent ligands with nickel or cobalt ions. Considerable improvements in catalytic properties have been accomplished through the incorporation of secondary coordination sphere interactions and, particularly, proton shuttle functionalities. Even though remarkably high catalytic performances have been achieved in some cases, the majority of these complexes only function in organic solvents and in the presence of strong acids, further highlighting the essential role of a bioinspired ligand framework in imparting enzyme-like properties.

Incorporation of catalytic centers into natural or designed protein scaffolds conceivably represents the most promising strategy to obtain artificial hydrogenases capable of paralleling their natural counterparts. Indeed, protein design methods allow precisely controlling the dielectric properties and the interactions within the residues in the active site, enabling a fine modulation of the metal redox properties. Furthermore, protein scaffolds can also serve as a platform to bind together catalytic and light-harvesting units, as reported in prominent examples discussed within the review.

In summary, biohybrid and bioinspired catalysts are promising candidates for sustainable hydrogen and energy production; nevertheless, some important challenges still need to be addressed. In particular, most artificial biomolecular catalysts are still fragile and display limited durability under operative conditions, especially in light-driven catalysis. One possible approach to overcome this problem is the immobilization of catalysts onto nanomaterials, which could extend their durability, allowing for easy catalyst recycling. Furthermore, as the majority of HECs reported so far contain transition metal ions such as nickel and cobalt, evaluation of their toxicity is a crucial aspect to consider, especially when scaling up the process to the industrial scale [277]. Indeed, the toxicity of metal complexes greatly depends on the nature of the ligands; thus, it is not trivial to predict [278]. A step further in the development of sustainable methods for hydrogen production would consist of the construction of completely protein or peptide-based materials, opening the way for the assembly of biosynthetic nanoreactors for energy-related catalysis.

## Figures and Tables

**Table 2:** Experimental catalytic parameters for photocatalytic hydrogen evolving systems discussed in the review.

Entry	Catalyst	Photosensitizer—Electron Donor	Solvent	pH	TON ^[a]^	TOF ^[b]^ (min^−1^)	Duration (h)	Ref.
1	[{(μSCH_2_)_2_NCH_2_C_6_H_5_}{Fe(CO)_3_} {Fe(CO)_2_P(Pyr)_3_}]	[Ir(ppy)_2_(bpy)]^+^—TEA	Acetone/H_2_O 9/1		466 (660)		8	[218]
2	[Fe_2_(μ-Cl_2_-bdt)(CO)_6_]	Ru(bpy)_3_^2+^—AscOH	H_2_O/DMF	5.5	200	3.5		[219]
3	[(μ-BDT)Fe_2_(CO)_6_]	Eosin Y—TEA	H_2_O/MeCN 1/1	10	404 (224)		4	[220]
4	Fe_2_S_2_-2SO_3_Na	Ru(bpy)_3_^2+^—AscOH	H_2_O	4.0	178	3.0	6	[221]
5	Fe_2_S_2_-SO_3_Na	Ru(bpy)_3_^2+^—AscOH	H_2_O	4.0	114	1.2	6	[221]
6	Fe_2_S_2_-2SO_3_Na	CdSe QDs—AscOH	H_2_O	4.0	2.65 × 10^4^	208	12	[221]
7	Fe_2_S_2_- SO_3_Na	CdSe QDs—AscOH	H_2_O	4.0	1.88 × 10^4^	127	12	[221]
8	{(μSCH_2_)2-silafluorene}{Fe_2_(CO)_6_}	1-silafluorene—TEA	MeCN		539	1.3	7	[222]
9	[Ni(P_2_^Ph^N_2_^Ph^)_2_]^2+^	Ru(bpy)_3_^2+^—AscOH	MeCN	2.25	2700	0.3	150	[223]
10	Ni(4,4′-OCH_3_-2,2′-bpy)(pyS)_2_	Fluorescein—TEA	H_2_O/EtOH 1/1	11.6	7300	5.2	30	[224]
11	[Ni(bpy)(mp)]	CdTe QDs—AscOH	H_2_O/DMF 2/1	4.5	6781	0.94	120	[123]
12	[Co(dmgBF_2_)_2_(OH_2_)_2_]	Ir(ppy)_2_(phen)]^+^—TEA	Acetone		165	0.88	15	[225]
13	[Co(dmgBF_2_)_2_(OH_2_)_2_]	[ReBr(CO)_3_(phen)]—TEA	Acetone		273	0.83	15	[225]
14	[Co(dmgH)_2_]	[Re(NCS)(bpy)(CO)_3_]—TEOA	DMF		1000 (6000)	0.93	16	[226]
15	[Co(dmgH)_2_pyCl]	[Pt(ttpy)(C≡CPh)]^+^—TEOA	MeCN/H_2_O 24/1	8.5	2150		10	[227]
16	CoP	Ru(bpy)_3_^2+^—AscO^−^	H_2_O	7	725	8.8	4.5	[228]
17	CoTPPS	Ru(bpy)_3_^2+^—AscO^−^	H_2_O	6.8	6410	120.8	1.5	[229]
18	[FeFe]-HSF	Ru(bpy)_3_^2+^—AscO^−^	H_2_O	7.4	8300 ^[c]^		3	[230]
19	[FeFe]-OVA	Ru(bpy)_3_^2+^—AscOH	H_2_O	5.3	35.6		3	[231]
20	[FeFe]-Cyt c	Ru(bpy)_3_^2+^—AscOH	H_2_O	4.7	82		2	[232]
21	[FeFe][Ru]-Pep18	Ru(bpy)(tpy)]^2+^—AscO^−^	H_2_O	8.5	9		2	[189]
22	[FeFe]-NB	Ru(bpy)_3_^2+^—AscOH	H_2_O	4.0	130	2.3	6	[94]
23	[FeFe]biot-SA	Ru(bpy)_3_^2+^—AscOH	H_2_O	4.5	47		1	[233]
24	[CoBr(appy)-Biot]-SA WT	Ru(bpy)_3_^2+^—AscOH	H_2_O	5	820		6	[234]
25	[CoBr(appy)-Biot]-SA S112K	Ru(bpy)_3_^2+^—AscOH	H_2_O	5	1070		6	[234]
26	Co-Mb	Ru(bpy)_3_^2+^—AscO^−^	H_2_O	7	518	1.47	8	[235]
27	Co-cyt b562 WT	Ru(bpy)_3_^2+^—AscO^−^	H_2_O	7	120		8	[236]
28	Co-cyt b562 M7D	Ru(bpy)_3_^2+^—AscO^−^	H_2_O	7	275		8	[236]
29	Co-cyt b562 M7A	Ru(bpy)_3_^2+^—AscO^−^	H_2_O	7	310		8	[236]
30	Co-αRep	Ru(bpy)_3_^2+^—AscO^−^	H_2_O	7	163		25	[237]
31	Co-MP11	Ru(bpy)_3_^2+^—TEOA	H_2_O	7.3	2390	7.0	20	[238]
32	Co-MC6*a	Ru(bpy)_3_^2+^—AscO^−^	H_2_O	6.5	10.4 × 10^4^	2.7	40	[239]
33	Cobaloxime-PSI	PSI—AscO^−^	H_2_O	6.3	5200	170	1.5	[240]
34	[Ni(P_2_^Ph^N_2_^Ph^)_2_]-PSI	PSI—AscO^−^	H_2_O	6.3	1870	44	3	[241]
35	Ni-ApoFld-PSI	PSI—AscO^−^	H_2_O	6.3	2825	75	4	[241]
36	Ru-Fd-Co	Ru(bpy)_3_^2+^—AscO^−^	H_2_O	6.3	320	1	6	[242]
37	RuNiRd	[Ru(bpy)2(5,6-epoxi-phen)]^2+^—AscO^−^	H_2_O	6.5	3.5		1.3	[243]
38	Ni-2SCC	Ru(bpy)_3_^2+^—AscOH	H_2_O	5.6	44	0.53	2	[244]
39	Ni-4SCC	Ru(bpy)_3_^2+^—AscOH	H_2_O	5.5	0.14		2	[245]

^[a]^ TON is calculated on the basis of the catalyst; values in parentheses are calculated on the basis of the photosensitizer. ^[b]^ TOF represents the maximum TOF (TON/min) assessed from initial rates of catalysis. ^[c]^ TON is calculated per single protein, containing multiple diiron catalysts. The TON relative to the single catalyst is 31.

## Data Availability

No new data were created or analyzed in this study. Data sharing is not applicable to this article.

## Abbreviations

2-amnt	2-aminobenzenethiolate
2SCC	two-stranded coiled coil
4-CNAnH^+^BF_4_^−^	4-cyanoanilinium tetrafluoroborate
4SCC	four-stranded coiled coil
(DO)(DOH)pn	(diacetylmonoximeimino)(diacetylmonoximateimino)-1,3-propane
AcOH	acetic acid
ADT	2-aza-propane-(1,3)-dithiolate
appy	bis-2,2″-bipyridine-6-yl(pyridine-2-yl)methanol
BA	benzoic acid
bdt	benezenedithiolate
BFC	biofuel cell
Biot	biotin
BNT	binaphtalenedithiolate
BO BPEC	bilirubin oxidase bio-photoelectrochemical cells
bpy	2,2′-bipyridine
Bu_4_N^+^PF_6_^−^	tetrabutylammonium hexafluorophosphate
Bz	benzyl
Bz-1-MeIm	benzyl-1-methylimidazolium
Cl8	2,3,7,8,12,13,17,18-octachloro- 5,10,15-tris(pentafluorophenyl)corrole
COF	covalent organic framework
Csp1	copper storage protein
dmgBF_2_	difluoroboryldimethylglyoximato
dmgH	dimethylglyoximato
DMF	dimethylformamide
dmnt	diaminomaleonitrile
DPE	dipeptide esther
Et_3_NH^+^BF_4_^−^	triethylammonium tetrafluoroborate
F15C	5,10,15-tris (pentafluorophenyl) corrole
FC	fuel cell
Fc-tpy	4′-ferrocenyl-2,2′:6′,2″-terpyridine
Fd	*Spinacia oleracea* ferredoxin
FFH	[FeFe]-hydrogenase
FH	[Fe]-hydrogenase
Fld GO	flavodoxin glucose oxidase
GP	guanylylpyridinol
HEC	Hydrogen-evolving catalyst
HER	hydrogen evolution reaction
Hmd	H_2_-forming methylenetetrahydromethanopterin dehydrogenases
HO	heme oxygenase
HSF	horse spleen ferritin
Ht-CoM61A	cobalt-substituted *Hydrogenobacter thermophilus* cytochrome c552
Im	imidazole
MC6*a	mimochrome VI*a
MeCN	acetonitrile
MP11	microperoxidase 11
mtim	2,6-bis-(1-methoxycarbonylmethyl-1h-1,2,3-triazol-4-yl)isonicotinate methyl ester
mp	2-hydroxythiophenol
NB	nitrobindin
NBP	nickel-binding protein
NFH	[NiFe]-hydrogenase
NFSH	[NiFeSe]-hydrogenase
NiRd	nickel-substituted *Desulfovibrio desulfuricans* rubredoxin
NiSOD	nickel superoxide dismutase
OEC	oxygen-evolving catalyst
OVA	ovalbumin
pbdt	2,2′-(propane-1,3-diylbis(sulfanediyl))dibenzenethiol)
PC	polycarbonate
PCET	proton-coupled electron transfer
pdt	propanedithiolate
PEC	photoelectrochemical cell
pfsc	5,10,15-tris(pentafluorophenyl)-2,17-bis(sulfonamide)corrole
phen	1,10-phenantroline
PPIX	protoporphyrin IX
Ppq	8-(1″,10″-phenanthrol-2″-yl)-2-(pyrid-2′-yl)quinoline
ppy	2-phenylpyridine
P(pyr)_3_	tris(N-pyrrolyl)phosphine
PSI	Photosystem I
PSII	Photosystem II
PT	proton transfer
ptim	2,6-bis(1-(pyridin-2-yl)-1h-1,2,3-triazol-4-yl)isonicotinate methyl ester
Py	pyridine
pyS	pyridine-2-thiolate
QD	quantum dot
SA	streptavidin
SAO	salicylketoxime
SDS	sodium dodecyl sulfate
SePh-PhO	phenyl selenoether phenolate
spmd	bis(salicylidene)-phenylmethanediamine
SwMb	sperm-whale myoglobin
tacn	1,4,7-triazacyclononane
TBA	tetrabutylamonium
tdas	1,2,5-thiadiazole-3,4-dithiolate
TEA	triethylamine
TEOA	triethanolamine
TFA	trifluoroacetic acid
TFE	2,2,2-trfifluoroethanol
TfO^−^	trifluoromethanesulfonate (triflate)
TFP	5,10,15,20-tetrakis(4-fluoro-2,6-dimethylphenyl)porphyrin
TOF	turnover frequency
TON	turnover number
TPPS	meso-tetra-(4-sulfonatophenyl)porphyrin
tpy	2,2′:6′,2″-terpyridine
TsOH	*p*-toluenesulfonic acid
ttpy	4′-*p*-tolylterpyridine

## References

[1] Lewis N.S., Nocera D.G. (2006). Powering the planet: Chemical challenges in solar energy utilization. Proc. Natl. Acad. Sci. USA.

[2] Sarkar O., Katakojwala R., Mohan S.V. (2021). Low carbon hydrogen production from a waste-based biorefinery system and environmental sustainability assessment. Green Chem..

[3] Borgschulte A. (2016). The Hydrogen Grand Challenge. Front. Energy Res..

[4] Pareek A., Dom R., Gupta J., Chandran J., Adepu V., Borse P.H. (2020). Insights into renewable hydrogen energy: Recent advances and prospects. Mater. Sci. Energy Technol..

[5] Wang T., Cao X., Jiao L. (2022). PEM water electrolysis for hydrogen production: Fundamentals, advances, and prospects. Carbon Neutrality.

[6] Shiva Kumar S., Himabindu V. (2019). Hydrogen production by PEM water electrolysis—A review. Mater. Sci. Energy Technol..

[7] Baeyens J., Zhang H., Nie J., Appels L., Dewil R., Ansart R., Deng Y. (2020). Reviewing the potential of bio-hydrogen production by fermentation. Renew. Sustain. Energy Rev..

[8] Li S., Li F., Zhu X., Liao Q., Chang J.-S., Ho S.-H. (2022). Biohydrogen production from microalgae for environmental sustainability. Chemosphere.

[9] Poladyan A., Margaryan L., Trchounian K., Trchounian A. (2020). Biomass and biohydrogen production during dark fermentation of *Escherichia coli* using office paper waste and cardboard. Int. J. Hydrog. Energy.

[10] Soares J.F., Confortin T.C., Todero I., Mayer F.D., Mazutti M.A. (2020). Dark fermentative biohydrogen production from lignocellulosic biomass: Technological challenges and future prospects. Renew. Sustain. Energy Rev..

[11] Arizzi M., Morra S., Gilardi G., Pugliese M., Gullino M.L., Valetti F. (2021). Improving sustainable hydrogen production from green waste: [FeFe]-hydrogenases quantitative gene expression RT-qPCR analysis in presence of autochthonous consortia. Biotechnol. Biofuels.

[12] Akkerman I., Janssen M., Rocha J., Wijffels R.H. (2002). Photobiological hydrogen production: Photochemical efficiency and bioreactor design. Int. J. Hydrog. Energy.

[13] Chen J., Li Q., Wang L., Fan C., Liu H. (2021). Advances in Whole-Cell Photobiological Hydrogen Production. Adv. NanoBiomed Res..

[14] Ghirardi M.L., Posewitz M.C., Maness P.-C., Dubini A., Yu J., Seibert M. (2007). Hydrogenases and Hydrogen Photoproduction in Oxygenic Photosynthetic Organisms. Annu. Rev. Plant Biol..

[15] Stripp S.T., Happe T. (2009). How algae produce hydrogen—News from the photosynthetic hydrogenase. Dalton Trans..

[16] Caserta G., Roy S., Atta M., Artero V., Fontecave M. (2015). Artificial hydrogenases: Biohybrid and supramolecular systems for catalytic hydrogen production or uptake. Curr. Opin. Chem. Biol..

[17] Holá K., Pavliuk M.V., Németh B., Huang P., Zdražil L., Land H., Berggren G., Tian H. (2020). Carbon Dots and [FeFe] Hydrogenase Biohybrid Assemblies for Efficient Light-Driven Hydrogen Evolution. ACS Catal..

[18] Martins M., Toste C., Pereira I.A.C. (2021). Enhanced Light-Driven Hydrogen Production by Self-Photosensitized Biohybrid Systems. Angew. Chem. Int. Ed..

[19] Lorenzi M., Gamache M.T., Redman H.J., Land H., Senger M., Berggren G. (2022). Light-Driven [FeFe] Hydrogenase Based H_2_ Production in *E. coli*: A Model Reaction for Exploring *E. coli* Based Semiartificial Photosynthetic Systems. ACS Sustain. Chem. Eng..

[20] Stripp S.T., Goldet G., Brandmayr C., Sanganas O., Vincent K.A., Haumann M., Armstrong F.A., Happe T. (2009). How oxygen attacks [FeFe] hydrogenases from photosynthetic organisms. Proc. Natl. Acad. Sci. USA.

[21] Simmons T.R., Berggren G., Bacchi M., Fontecave M., Artero V. (2014). Mimicking hydrogenases: From biomimetics to artificial enzymes. Coord. Chem. Rev..

[22] Amaro-Gahete J., Pavliuk M.V., Tian H., Esquivel D., Romero-Salguero F.J., Ott S. (2021). Catalytic systems mimicking the [FeFe]-hydrogenase active site for visible-light-driven hydrogen production. Coord. Chem. Rev..

[23] Lubitz W., Ogata H., Rüdiger O., Reijerse E. (2014). Hydrogenases. Chem. Rev..

[24] Greening C., Cook G.M. (2014). Integration of hydrogenase expression and hydrogen sensing in bacterial cell physiology. Curr. Opin. Microbiol..

[25] Lu Y., Koo J. (2019). O_2_ sensitivity and H_2_ production activity of hydrogenases—A review. Biotechnol. Bioeng..

[26] Morra S. (2022). Fantastic [FeFe]-Hydrogenases and Where to Find Them. Front. Microbiol..

[27] Peters J.W., Schut G.J., Boyd E.S., Mulder D.W., Shepard E.M., Broderick J.B., King P.W., Adams M.W.W. (2015). [FeFe]- and [NiFe]-hydrogenase diversity, mechanism, and maturation. Biochim. Biophys. Acta BBA Mol. Cell Res..

[28] Poudel S., Tokmina-Lukaszewska M., Colman D.R., Refai M., Schut G.J., King P.W., Maness P.-C., Adams M.W.W., Peters J.W., Bothner B. (2016). Unification of [FeFe]-hydrogenases into three structural and functional groups. Biochim. Biophys. Acta BBA Gen. Subj..

[29] Wittkamp F., Senger M., Stripp S.T., Apfel U.-P. (2018). [FeFe]-Hydrogenases: Recent developments and future perspectives. Chem. Commun..

[30] Beinert H. (2000). Iron-sulfur proteins: Ancient structures, still full of surprises. JBIC J. Biol. Inorg. Chem..

[31] Stiebritz M.T., Reiher M. (2012). Hydrogenases and oxygen. Chem. Sci..

[32] Rodríguez-Maciá P., Galle L.M., Bjornsson R., Lorent C., Zebger I., Yoda Y., Cramer S.P., DeBeer S., Span I., Birrell J.A. (2020). Caught in the H_inact_: Crystal Structure and Spectroscopy Reveal a Sulfur Bound to the Active Site of an O_2_ -stable State of [FeFe] Hydrogenase. Angew. Chem. Int. Ed..

[33] Rodríguez-Maciá P., Reijerse E.J., van Gastel M., DeBeer S., Lubitz W., Rüdiger O., Birrell J.A. (2018). Sulfide Protects [FeFe] Hydrogenases From O_2_. J. Am. Chem. Soc..

[34] Völler J.-S. (2018). Air-stable [FeFe] hydrogenases. Nat. Catal..

[35] Birrell J.A., Rodríguez-Maciá P., Reijerse E.J., Martini M.A., Lubitz W. (2021). The catalytic cycle of [FeFe] hydrogenase: A tale of two sites. Coord. Chem. Rev..

[36] Land H., Senger M., Berggren G., Stripp S.T. (2020). Current State of [FeFe]-Hydrogenase Research: Biodiversity and Spectroscopic Investigations. ACS Catal..

[37] Vignais P.M., Billoud B., Meyer J. (2001). Classification and phylogeny of hydrogenases1. FEMS Microbiol. Rev..

[38] Alfano M., Cavazza C. (2020). Structure, function, and biosynthesis of nickel-dependent enzymes. Protein Sci..

[39] Benoit S.L., Maier R.J., Sawers R.G., Greening C. (2020). Molecular Hydrogen Metabolism: A Widespread Trait of Pathogenic Bacteria and Protists. Microbiol. Mol. Biol. Rev..

[40] Greening C., Biswas A., Carere C.R., Jackson C.J., Taylor M.C., Stott M.B., Cook G.M., Morales S.E. (2016). Genomic and metagenomic surveys of hydrogenase distribution indicate H_2_ is a widely utilised energy source for microbial growth and survival. ISME J..

[41] Garcin E., Vernede X., Hatchikian E., Volbeda A., Frey M., Fontecilla-Camps J. (1999). The crystal structure of a reduced [NiFeSe] hydrogenase provides an image of the activated catalytic center. Structure.

[42] Wombwell C., Caputo C.A., Reisner E. (2015). [NiFeSe]-Hydrogenase Chemistry. Acc. Chem. Res..

[43] Radu V., Frielingsdorf S., Lenz O., Jeuken L.J.C. (2016). Reactivation from the Ni–B state in [NiFe] hydrogenase of Ralstonia eutropha is controlled by reduction of the superoxidised proximal cluster. Chem. Commun..

[44] Lukey M.J., Roessler M.M., Parkin A., Evans R.M., Davies R.A., Lenz O., Friedrich B., Sargent F., Armstrong F.A. (2011). Oxygen-Tolerant [NiFe]-Hydrogenases: The Individual and Collective Importance of Supernumerary Cysteines at the Proximal Fe-S Cluster. J. Am. Chem. Soc..

[45] Ogata H., Lubitz W., Higuchi Y. (2016). Structure and function of [NiFe] hydrogenases. J. Biochem..

[46] Evans R.M., Brooke E.J., Wehlin S.A.M., Nomerotskaia E., Sargent F., Carr S.B., Phillips S.E.V., Armstrong F.A. (2016). Mechanism of hydrogen activation by [NiFe] hydrogenases. Nat. Chem. Biol..

[47] Ogata H., Lubitz W., Higuchi Y. (2009). [NiFe] hydrogenases: Structural and spectroscopic studies of the reaction mechanism. Dalton Trans..

[48] Tai H., Hirota S. (2020). Mechanism and Application of the Catalytic Reaction of [NiFe] Hydrogenase: Recent Developments. ChemBioChem.

[49] Siegbahn P.E.M. (2021). A quantum chemical approach for the mechanisms of redox-active metalloenzymes. RSC Adv..

[50] Siegbahn P.E.M., Liao R.-Z. (2020). The Energetics of Hydrogen Molecule Oxidation in NiFe-Hydrogenase. ACS Catal..

[51] Wang C., Lai Z., Huang G., Pan H.-J. (2022). Current State of [Fe]-Hydrogenase and Its Biomimetic Models. Chem. Eur. J..

[52] Huang G., Wagner T., Wodrich M.D., Ataka K., Bill E., Ermler U., Hu X., Shima S. (2019). The atomic-resolution crystal structure of activated [Fe]-hydrogenase. Nat. Catal..

[53] Shima S., Lyon E.J., Sordel-Klippert M., Kauß M., Kahnt J., Thauer R.K., Steinbach K., Xie X., Verdier L., Griesinger C. (2004). The Cofactor of the Iron–Sulfur Cluster Free Hydrogenase Hmd: Structure of the Light-Inactivation Product. Angew. Chem. Int. Ed..

[54] Tamura H., Salomone-Stagni M., Fujishiro T., Warkentin E., Meyer-Klaucke W., Ermler U., Shima S. (2013). Crystal Structures of [Fe]-Hydrogenase in Complex with Inhibitory Isocyanides: Implications for the H2-Activation Site. Angew. Chem. Int. Ed..

[55] Hiromoto T., Ataka K., Pilak O., Vogt S., Stagni M.S., Meyer-Klaucke W., Warkentin E., Thauer R.K., Shima S., Ermler U. (2009). The crystal structure of C176A mutated [Fe]-hydrogenase suggests an acyl-iron ligation in the active site iron complex. FEBS Lett..

[56] Yang X., Hall M.B. (2009). Monoiron Hydrogenase Catalysis: Hydrogen Activation with the Formation of a Dihydrogen, Fe−Hδ−···Hδ+−O, Bond and Methenyl-H4MPT+ Triggered Hydride Transfer. J. Am. Chem. Soc..

[57] Hedegård E.D., Kongsted J., Ryde U. (2015). Multiscale Modeling of the Active Site of [Fe] Hydrogenase: The H2 Binding Site in Open and Closed Protein Conformations. Angew. Chem. Int. Ed..

[58] Ruth J.C., Spormann A.M. (2021). Enzyme Electrochemistry for Industrial Energy Applications—A Perspective on Future Areas of Focus. ACS Catal..

[59] Ruff A., Conzuelo F., Schuhmann W. (2020). Bioelectrocatalysis as the basis for the design of enzyme-based biofuel cells and semi-artificial biophotoelectrodes. Nat. Catal..

[60] Liu X., Risbakk S., Almeida Carvalho P., Yang M., Hoff Backe P., Bjørås M., Norby T., Chatzitakis A. (2022). Immobilization of FeFe-hydrogenase on black TiO_2_ nanotubes as biocathodes for the hydrogen evolution reaction. Electrochem. Commun..

[61] Ruff A., Szczesny J., Vega M., Zacarias S., Matias P.M., Gounel S., Mano N., Pereira I.A.C., Schuhmann W. (2020). Redox-Polymer-Wired [NiFeSe] Hydrogenase Variants with Enhanced O2 Stability for Triple-Protected High-Current-Density H2-Oxidation Bioanodes. ChemSusChem.

[62] Shiraiwa S., So K., Sugimoto Y., Kitazumi Y., Shirai O., Nishikawa K., Higuchi Y., Kano K. (2018). Reactivation of standard [NiFe]-hydrogenase and bioelectrochemical catalysis of proton reduction and hydrogen oxidation in a mediated-electron-transfer system. Bioelectrochemistry.

[63] Oughli A.A., Vélez M., Birrell J.A., Schuhmann W., Lubitz W., Plumeré N., Rüdiger O. (2018). Viologen-modified electrodes for protection of hydrogenases from high potential inactivation while performing H_2_ oxidation at low overpotential. Dalton Trans..

[64] Olloqui-Sariego J.L., Calvente J.J., Andreu R. (2021). Immobilizing redox enzymes at mesoporous and nanostructured electrodes. Curr. Opin. Electrochem..

[65] Walter M.G., Warren E.L., McKone J.R., Boettcher S.W., Mi Q., Santori E.A., Lewis N.S. (2010). Solar Water Splitting Cells. Chem. Rev..

[66] Li J., Wu N. (2015). Semiconductor-based photocatalysts and photoelectrochemical cells for solar fuel generation: A review. Catal. Sci. Technol..

[67] Hagfeldt A., Boschloo G., Sun L., Kloo L., Pettersson H. (2010). Dye-Sensitized Solar Cells. Chem. Rev..

[68] Li Z., Wang W., Ding C., Wang Z., Liao S., Li C. (2017). Biomimetic electron transport via multiredox shuttles from photosystem II to a photoelectrochemical cell for solar water splitting. Energy Environ. Sci..

[69] Du J., Xin J., Liu M., Zhang X., He H., Wu J., Xu X. (2022). Preparation of Photo-Bioelectrochemical Cells With the RC-LH Complex From Roseiflexus castenholzii. Front. Microbiol..

[70] Zhang H., Sun X., Hao S., Dong S. (2022). A solar-rechargeable bio-photoelectrochemical system based on carbon tracking strategy for enhancement of glucose electrometabolism. Nano Energy.

[71] Mukha D., Cohen Y., Yehezkeli O. (2020). Bismuth Vanadate/Bilirubin Oxidase Photo(bio)electrochemical Cells for Unbiased, Light-Triggered Electrical Power Generation. ChemSusChem.

[72] Huang X., Ren L., Jiang C., Han X., Yin X., Liu Y., Yang W., Chen Y. (2022). Design of a novel photoelectrochemical enzymatic biofuel cell with high power output under visible light. Chem. Eng. J..

[73] Pinhassi R.I., Kallmann D., Saper G., Dotan H., Linkov A., Kay A., Liveanu V., Schuster G., Adir N., Rothschild A. (2016). Hybrid bio-photo-electro-chemical cells for solar water splitting. Nat. Commun..

[74] Sokol K.P., Robinson W.E., Warnan J., Kornienko N., Nowaczyk M.M., Ruff A., Zhang J.Z., Reisner E. (2018). Bias-free photoelectrochemical water splitting with photosystem II on a dye-sensitized photoanode wired to hydrogenase. Nat. Energy.

[75] Mersch D., Lee C.-Y., Zhang J.Z., Brinkert K., Fontecilla-Camps J.C., Rutherford A.W., Reisner E. (2015). Wiring of Photosystem II to Hydrogenase for Photoelectrochemical Water Splitting. J. Am. Chem. Soc..

[76] Yates N.D.J., Fascione M.A., Parkin A. (2018). Methodologies for “Wiring” Redox Proteins/Enzymes to Electrode Surfaces. Chem. Eur. J..

[77] Waterhouse G.I.N., Waterland M.R. (2007). Opal and inverse opal photonic crystals: Fabrication and characterization. Polyhedron.

[78] Fathi F., Monirinasab H., Ranjbary F., Nejati-Koshki K. (2022). Inverse opal photonic crystals: Recent advances in fabrication methods and biological applications. J. Drug Deliv. Sci. Technol..

[79] Nam D.H., Zhang J.Z., Andrei V., Kornienko N., Heidary N., Wagner A., Nakanishi K., Sokol K.P., Slater B., Zebger I. (2018). Solar Water Splitting with a Hydrogenase Integrated in Photoelectrochemical Tandem Cells. Angew. Chem. Int. Ed..

[80] Park N.-G. (2015). Perovskite solar cells: An emerging photovoltaic technology. Mater. Today.

[81] Wu T., Qin Z., Wang Y., Wu Y., Chen W., Zhang S., Cai M., Dai S., Zhang J., Liu J. (2021). The Main Progress of Perovskite Solar Cells in 2020–2021. Nano-Micro Lett..

[82] Edwardes Moore E., Andrei V., Zacarias S., Pereira I.A.C., Reisner E. (2020). Integration of a Hydrogenase in a Lead Halide Perovskite Photoelectrode for Tandem Solar Water Splitting. ACS Energy Lett..

[83] Tian L., Németh B., Berggren G., Tian H. (2018). Hydrogen evolution by a photoelectrochemical cell based on a Cu_2_O-ZnO-[FeFe] hydrogenase electrode. J. Photochem. Photobiol. Chem..

[84] Mazurenko I., Wang X., de Poulpiquet A., Lojou E. (2017). H_2_/O_2_ enzymatic fuel cells: From proof-of-concept to powerful devices. Sustain. Energy Fuels.

[85] Gentil S., Che Mansor S.M., Jamet H., Cosnier S., Cavazza C., Le Goff A. (2018). Oriented Immobilization of [NiFeSe] Hydrogenases on Covalently and Noncovalently Functionalized Carbon Nanotubes for H_2_/Air Enzymatic Fuel Cells. ACS Catal..

[86] Hardt S., Stapf S., Filmon D.T., Birrell J.A., Rüdiger O., Fourmond V., Léger C., Plumeré N. (2021). Reversible H_2_ oxidation and evolution by hydrogenase embedded in a redox polymer film. Nat. Catal..

[87] Szczesny J., Marković N., Conzuelo F., Zacarias S., Pereira I.A.C., Lubitz W., Plumeré N., Schuhmann W., Ruff A. (2018). A gas breathing hydrogen/air biofuel cell comprising a redox polymer/hydrogenase-based bioanode. Nat. Commun..

[88] Szczesny J., Birrell J.A., Conzuelo F., Lubitz W., Ruff A., Schuhmann W. (2020). Redox-Polymer-Based High-Current-Density Gas-Diffusion H_2_-Oxidation Bioanode Using [FeFe] Hydrogenase from Desulfovibrio desulfuricans in a Membrane-Free Biofuel Cell. Angew. Chem. Int. Ed..

[89] Wang Y., Song Y., Ma C., Kang Z., Zhu Z. (2021). A heterologously-expressed thermostable Pyrococcus furiosus cytoplasmic [NiFe]-hydrogenase I used as the catalyst of H_2_/air biofuel cells. Int. J. Hydrog. Energy.

[90] Wu H., Feng C., Zhang L., Zhang J., Wilkinson D.P. (2021). Non-Noble Metal Electrocatalysts for the Hydrogen Evolution Reaction in Water Electrolysis. Electrochem. Energy Rev..

[91] Zou X., Zhang Y. (2015). Noble metal-free hydrogen evolution catalysts for water splitting. Chem. Soc. Rev..

[92] Eftekhari A. (2017). Electrocatalysts for hydrogen evolution reaction. Int. J. Hydrog. Energy.

[93] Ahmed M.E., Nayek A., Križan A., Coutard N., Morozan A., Ghosh Dey S., Lomoth R., Hammarström L., Artero V., Dey A. (2022). A Bidirectional Bioinspired [FeFe]-Hydrogenase Model. J. Am. Chem. Soc..

[94] Onoda A., Kihara Y., Fukumoto K., Sano Y., Hayashi T. (2014). Photoinduced Hydrogen Evolution Catalyzed by a Synthetic Diiron Dithiolate Complex Embedded within a Protein Matrix. ACS Catal..

[95] Gao S., Liu Y., Shao Y., Jiang D., Duan Q. (2020). Iron carbonyl compounds with aromatic dithiolate bridges as organometallic mimics of [FeFe] hydrogenases. Coord. Chem. Rev..

[96] Marr A.C., Spencer D.J.E., Schröder M. (2001). Structural mimics for the active site of [NiFe] hydrogenase. Coord. Chem. Rev..

[97] Ahmed M.E., Dey A. (2019). Recent developments in bioinspired modelling of [NiFe]- and [FeFe]-hydrogenases. Curr. Opin. Electrochem..

[98] Dey S., Das P.K., Dey A. (2013). Mononuclear iron hydrogenase. Coord. Chem. Rev..

[99] Wang M., Chen L., Li X., Sun L. (2011). Approaches to efficient molecular catalyst systems for photochemical H_2_ production using [FeFe]-hydrogenase active site mimics. Dalton Trans..

[100] Gómez-Gallego M., Sierra M.A. (2021). Deuteration mechanistic studies of hydrogenase mimics. Inorg. Chem. Front..

[101] Schmidt M., Contakes S.M., Rauchfuss T.B. (1999). First Generation Analogues of the Binuclear Site in the Fe-Only Hydrogenases:  Fe_2_(μ-SR)_2_(CO)_4_(CN)_2_^2-^. J. Am. Chem. Soc..

[102] Lyon E.J., Georgakaki I.P., Reibenspies J.H., Darensbourg M.Y. (1999). Carbon Monoxide and Cyanide Ligands in a Classical Organometallic Complex Model for Fe-Only Hydrogenase. Angew. Chem. Int. Ed..

[103] Cloirec A.L., Davies S.C., Evans D.J., Hughes D.L., Pickett C.J., Best S.P., Borg S. (1999). A di-iron dithiolate possessing structural elements of the carbonyl/cyanide sub-site of the H-centre of Fe-only hydrogenase. Chem. Commun..

[104] Song L.-C., Yang Z.-Y., Bian H.-Z., Hu Q.-M. (2004). Novel Single and Double Diiron Oxadithiolates as Models for the Active Site of [Fe]-Only Hydrogenases. Organometallics.

[105] Lawrence J.D., Li H., Rauchfuss T.B., Bénard M., Rohmer M.-M. (2001). Diiron Azadithiolates as Models for the Iron-Only Hydrogenase Active Site: Synthesis, Structure, and Stereoelectronics. Angew. Chem. Int. Ed..

[106] Karnahl M., Tschierlei S., Erdem Ö.F., Pullen S., Santoni M.-P., Reijerse E.J., Lubitz W., Ott S. (2012). Mixed-valence [FeIFeII] hydrogenase active site model complexes stabilized by a bidentate carborane bis-phosphine ligand. Dalton Trans..

[107] Kositzki R., Mebs S., Schuth N., Leidel N., Schwartz L., Karnahl M., Wittkamp F., Daunke D., Grohmann A., Apfel U.-P. (2017). Electronic and molecular structure relations in diiron compounds mimicking the [FeFe]-hydrogenase active site studied by X-ray spectroscopy and quantum chemistry. Dalton Trans..

[108] Arrigoni F., Bertini L., Breglia R., Greco C., Gioia L.D., Zampella G. (2020). Catalytic H_2_ evolution/oxidation in [FeFe]-hydrogenase biomimetics: Account from DFT on the interplay of related issues and proposed solutions. New J. Chem..

[109] Natarajan M., Kumar N., Joshi M., Stein M., Kaur-Ghumaan S. (2022). Mechanism of Diiron Hydrogenase Complexes Controlled by Nature of Bridging Dithiolate Ligand. ChemistryOpen.

[110] Morvan D., Capon J.-F., Gloaguen F., Le Goff A., Marchivie M., Michaud F., Schollhammer P., Talarmin J., Yaouanc J.-J., Pichon R. (2007). N-Heterocyclic Carbene Ligands in Nonsymmetric Diiron Models of Hydrogenase Active Sites. Organometallics.

[111] Chouffai D., Zampella G., Capon J.-F., Gioia L.D., Goff A.L., Pétillon F.Y., Schollhammer P., Talarmin J. (2012). Electrochemical and Theoretical Studies of the Impact of the Chelating Ligand on the Reactivity of [Fe_2_(CO)_4_(κ^2^-LL)(μ-pdt)]^+^ Complexes with Different Substrates (LL = I_Me_-CH_2_-I_Me_, dppe; I_Me_ = 1-Methylimidazol-2-ylidene). Organometallics.

[112] Erdem Ö.F., Stein M., Kaur-Ghumaan S., Reijerse E.J., Ott S., Lubitz W. (2013). Effect of Cyanide Ligands on the Electronic Structure of [FeFe] Hydrogenase Active-Site Model Complexes with an Azadithiolate Cofactor. Chem. Eur. J..

[113] Schwartz L., Singh P.S., Eriksson L., Lomoth R., Ott S. (2008). Tuning the electronic properties of Fe_2_(μ-arenedithiolate)(CO)_6−n_(PMe_3_)_n_ (n = 0, 2) complexes related to the [Fe–Fe]-hydrogenase active site. Comptes Rendus Chim..

[114] Donovan E.S., McCormick J.J., Nichol G.S., Felton G.A.N. (2012). Cyclic Voltammetric Studies of Chlorine-Substituted Diiron Benzenedithiolato Hexacarbonyl Electrocatalysts Inspired by the [FeFe]-Hydrogenase Active Site. Organometallics.

[115] McKone J.R., Marinescu S.C., Brunschwig B.S., Winkler J.R., Gray H.B. (2014). Earth-abundant hydrogen evolution electrocatalysts. Chem. Sci..

[116] Drosou M., Kamatsos F., Mitsopoulou C.A. (2020). Recent advances in the mechanisms of the hydrogen evolution reaction by non-innocent sulfur-coordinating metal complexes. Inorg. Chem. Front..

[117] Pitchaimani J., Ni S.-F., Dang L. (2020). Metal dithiolene complexes in olefin addition and purification, small molecule adsorption, H_2_ evolution and CO_2_ reduction. Coord. Chem. Rev..

[118] Singh B., Indra A. (2020). Role of redox active and redox non-innocent ligands in water splitting. Inorganica Chim. Acta.

[119] Niu Z., Yang L., Xiao Y., Xue M., Zhou J., Zhang L., Zhang J., Wilkinson D.P., Ni C. (2022). Novel Dithiolene Nickel Complex Catalysts for Electrochemical Hydrogen Evolution Reaction for Hydrogen Production in Nonaqueous and Aqueous Solutions. Electrocatalysis.

[120] Drosou M., Zarkadoulas A., Bethanis K., Mitsopoulou C.A. (2021). Structural modifications on nickel dithiolene complexes lead to increased metal participation in the electrocatalytic hydrogen evolution mechanism. J. Coord. Chem..

[121] Yin H.-J., Wang Z., Zhao Z.-Y., Jiang X.-Y., Yu J.-Y., Yang L.-M., Zhang Y.-M., Liu W., Ni C.-L. (2023). Synthesis, crystal structure and properties of electro-catalysis for hydrogen production of a molecular nickel catalyst based on bis(1,2,5-thiadiazole-3,4-dithiolate) ligand. J. Mol. Struct..

[122] Kamatsos F., Drosou M., Mitsopoulou C.A. (2021). Heteroleptic thiolate diamine nickel complexes: Noble-free-metal catalysts in electrocatalytic and light-driven hydrogen evolution reaction. Int. J. Hydrog. Energy.

[123] Kamatsos F., Bethanis K., Mitsopoulou C.A. (2021). Synthesis of Novel Heteroleptic Oxothiolate Ni(II) Complexes and Evaluation of Their Catalytic Activity for Hydrogen Evolution. Catalysts.

[124] Chen L., Xie B., Li T., Lai C., Cao J.-X., Ji R.-W., Liu M.-N., Li W., Zhang D.-L., He J.-Y. (2022). Heteroleptic nickel complexes bearing O-methyldithiophosphate and aminodiphosphine monosulfide ligands as robust molecular electrocatalysts for hydrogen evolution. Appl. Organomet. Chem..

[125] Artero V., Chavarot-Kerlidou M., Fontecave M. (2011). Splitting Water with Cobalt. Angew. Chem. Int. Ed..

[126] Dolui D., Ghorai S., Dutta A. (2020). Tuning the reactivity of cobalt-based H_2_ production electrocatalysts via the incorporation of the peripheral basic functionalities. Coord. Chem. Rev..

[127] McCrory C.C.L., Uyeda C., Peters J.C. (2012). Electrocatalytic hydrogen evolution in acidic water with molecular cobalt tetraazamacrocycles. J. Am. Chem. Soc..

[128] Sun D., Karippara Harshan A., Pécaut J., Hammes-Schiffer S., Costentin C., Artero V. (2021). Hydrogen Evolution Mediated by Cobalt Diimine-Dioxime Complexes: Insights into the Role of the Ligand Acid/Base Functionalities. ChemElectroChem.

[129] Wang C.-L., Yang H., Du J., Zhan S.-Z. (2021). Catalytic performance of a square planar nickel complex for electrochemical- and photochemical-driven hydrogen evolution from water. Inorg. Chem. Commun..

[130] Liu J., Liao R.-Z., Heinemann F.W., Meyer K., Thummel R.P., Zhang Y., Tong L. (2021). Electrocatalytic Hydrogen Evolution by Cobalt Complexes with a Redox Non-Innocent Polypyridine Ligand. Inorg. Chem..

[131] Kumar Padhi S., Ahmad E., Rai S., Panda B. (2020). Kinetics and mechanistic study of electrocatalytic hydrogen evolution by [Co(Fc-tpy)_2_]^2+^. Polyhedron.

[132] Cui H.-B., Li J.-H., Zhang X., Zhou M., Huang Z.-Z., Lai Y.-C., Qiu J.-X., Ren Y.-J., Zhang H.-X. (2022). Electrocatalytic hydrogen evolution by Co(II) complexes of bistriazolylpyridines. Int. J. Hydrog. Energy.

[133] Upadhyay A., Meena H., Kumar Jha R., Kanika, Kumar S. (2022). Isolation of monomeric copper(ii) phenolate selenoether complexes using chelating ortho -bisphenylselenide-phenolate ligands and their electrocatalytic hydrogen gas evolution activity. Dalton Trans..

[134] To T.H., Tran D.B., Ha V.T.T., Tran P.D. (2022). Electrocatalytic H_2_ evolution using binuclear cobalt complexes as catalysts. RSC Adv..

[135] Chou P., Kim L., Marzouk S.M., Sun R., Hartnett A.C., Dogutan D.K., Zheng S.-L., Nocera D.G. (2022). Synthesis, Characterization, and Hydrogen Evolution Activity of Metallo-meso-(4-fluoro-2,6-dimethylphenyl)porphyrin Derivatives. ACS Omega.

[136] Chen Q.-C., Fite S., Fridman N., Tumanskii B., Mahammed A., Gross Z. (2022). Hydrogen Evolution Catalyzed by Corrole-Chelated Nickel Complexes, Characterized in All Catalysis-Relevant Oxidation States. ACS Catal..

[137] Wan B., Cheng F., Lan J., Zhao Y., Yang G., Sun Y.-M., Si L.-P., Liu H.-Y. (2022). Electrocatalytic hydrogen evolution of manganese corrole. Int. J. Hydrog. Energy.

[138] Sudhakar K., Panda P.K. (2022). Tuning Proton Reduction Efficiencies of Copper Corrole in Electrocatalysis via Multiple β-Chloro Substitution. ACS Appl. Energy Mater..

[139] Smith S.E., Yang J.Y., DuBois D.L., Bullock R.M. (2012). Reversible Electrocatalytic Production and Oxidation of Hydrogen at Low Overpotentials by a Functional Hydrogenase Mimic. Angew. Chem. Int. Ed..

[140] Jacobsen G.M., Yang J.Y., Twamley B., Wilson A.D., Bullock R.M., DuBois M.R., DuBois D.L. (2008). Hydrogen production using cobalt-based molecular catalysts containing a proton relay in the second coordination sphere. Energy Environ. Sci..

[141] Ahmed M.E., Dey S., Darensbourg M.Y., Dey A. (2018). Oxygen-Tolerant H_2_ Production by [FeFe]-H_2ase_ Active Site Mimics Aided by Second Sphere Proton Shuttle. J. Am. Chem. Soc..

[142] Li X., Lv B., Zhang X.-P., Jin X., Guo K., Zhou D., Bian H., Zhang W., Apfel U.-P., Cao R. (2022). Introducing Water-Network-Assisted Proton Transfer for Boosted Electrocatalytic Hydrogen Evolution with Cobalt Corrole. Angew. Chem. Int. Ed..

[143] Quentel F., Passard G., Gloaguen F. (2012). Electrochemical hydrogen production in aqueous micellar solution by a diiron benzenedithiolate complex relevant to [FeFe] hydrogenases. Energy Environ. Sci..

[144] Nurttila S.S., Zaffaroni R., Mathew S., Reek J.N.H. (2019). Control of the overpotential of a [FeFe] hydrogenase mimic by a synthetic second coordination sphere. Chem. Commun..

[145] Jain A., Lense S., Linehan J.C., Raugei S., Cho H., DuBois D.L., Shaw W.J. (2011). Incorporating Peptides in the Outer-Coordination Sphere of Bioinspired Electrocatalysts for Hydrogen Production. Inorg. Chem..

[146] Kleingardner J.G., Kandemir B., Bren K.L. (2014). Hydrogen Evolution from Neutral Water under Aerobic Conditions Catalyzed by Cobalt Microperoxidase-11. J. Am. Chem. Soc..

[147] Kandemir B., Chakraborty S., Guo Y., Bren K.L. (2016). Semisynthetic and Biomolecular Hydrogen Evolution Catalysts. Inorg. Chem..

[148] Firpo V., Le J.M., Pavone V., Lombardi A., Bren K.L. (2018). Hydrogen evolution from water catalyzed by cobalt-mimochrome VI*a, a synthetic mini-protein. Chem. Sci..

[149] Bacchi M., Berggren G., Niklas J., Veinberg E., Mara M.W., Shelby M.L., Poluektov O.G., Chen L.X., Tiede D.M., Cavazza C. (2014). Cobaloxime-Based Artificial Hydrogenases. Inorg. Chem..

[150] Bacchi M., Veinberg E., Field M.J., Niklas J., Matsui T., Tiede D.M., Poluektov O.G., Ikeda-Saito M., Fontecave M., Artero V. (2016). Artificial Hydrogenases Based on Cobaloximes and Heme Oxygenase. ChemPlusChem.

[151] Call A., Casadevall C., Romero-Rivera A., Martin-Diaconescu V., Sommer D.J., Osuna S., Ghirlanda G., Lloret-Fillol J. (2019). Improved Electro- and Photocatalytic Water Reduction by Confined Cobalt Catalysts in Streptavidin. ACS Catal..

[152] Slater J.W., Shafaat H.S. (2015). Nickel-Substituted Rubredoxin As a Minimal Enzyme Model for Hydrogenase. J. Phys. Chem. Lett..

[153] Slater J.W., Marguet S.C., Gray M.E., Monaco H.A., Sotomayor M., Shafaat H.S. (2019). Power of the Secondary Sphere: Modulating Hydrogenase Activity in Nickel-Substituted Rubredoxin. ACS Catal..

[154] Selvan D., Prasad P., Farquhar E.R., Shi Y., Crane S., Zhang Y., Chakraborty S. (2019). Redesign of a Copper Storage Protein into an Artificial Hydrogenase. ACS Catal..

[155] Drosou M., Kamatsos F., Ioannidis G., Zarkadoulas A., Mitsopoulou C.A., Papatriantafyllopoulou C., Tzeli D. (2020). Reactivity and Mechanism of Photo- and Electrocatalytic Hydrogen Evolution by a Diimine Copper(I) Complex. Catalysts.

[156] Tong L., Duan L., Zhou A., Thummel R.P. (2020). First-row transition metal polypyridine complexes that catalyze proton to hydrogen reduction. Coord. Chem. Rev..

[157] Droghetti F., Lucarini F., Molinari A., Ruggi A., Natali M. (2022). Recent findings and future directions in photosynthetic hydrogen evolution using polypyridine cobalt complexes. Dalton Trans..

[158] Agarwal T., Kaur-Ghumaan S. (2019). HER catalysed by iron complexes without a Fe_2_S_2_ core: A review. Coord. Chem. Rev..

[159] Beyene B.B., Hung C.-H. (2020). Recent progress on metalloporphyrin-based hydrogen evolution catalysis. Coord. Chem. Rev..

[160] Li X., Lei H., Xie L., Wang N., Zhang W., Cao R. (2022). Metalloporphyrins as Catalytic Models for Studying Hydrogen and Oxygen Evolution and Oxygen Reduction Reactions. Acc. Chem. Res..

[161] O’Neill J.S., Kearney L., Brandon M.P., Pryce M.T. (2022). Design components of porphyrin-based photocatalytic hydrogen evolution systems: A review. Coord. Chem. Rev..

[162] Castro-Cruz H.M., Macías-Ruvalcaba N.A. (2022). Porphyrin-catalyzed electrochemical hydrogen evolution reaction. Metal-centered and ligand-centered mechanisms. Coord. Chem. Rev..

[163] Liang Y.-Y., Li M.-Y., Shi L., Lin D.-Z., Zhan S.-Z., Liu H.-Y. (2021). Electrocatalytic hydrogen evolution by cobalt triaryl corroles with appended ester and carboxyl on the 10-phenyl group. J. Coord. Chem..

[164] Artero V., Fontecave M. (2005). Some general principles for designing electrocatalysts with hydrogenase activity. Coord. Chem. Rev..

[165] Collman J.P., Ha Y., Wagenknecht P.S., Lopez M.A., Guilard R. (1993). Cofacial bisorganometallic diporphyrins: Synthetic control in proton reduction catalysis. J. Am. Chem. Soc..

[166] Rauchfuss T.B. (2015). Diiron Azadithiolates As Models for the [FeFe]-Hydrogenase Active Site and Paradigm for the Role of the Second Coordination Sphere. Acc. Chem. Res..

[167] Thammavongsy Z., Mercer I.P., Yang J.Y. (2019). Promoting proton coupled electron transfer in redox catalysts through molecular design. Chem. Commun..

[168] Tyburski R., Liu T., Glover S.D., Hammarström L. (2021). Proton-Coupled Electron Transfer Guidelines, Fair and Square. J. Am. Chem. Soc..

[169] DuBois M.R., DuBois D.L. (2008). The roles of the first and second coordination spheres in the design of molecular catalysts for H_2_ production and oxidation. Chem. Soc. Rev..

[170] Wiedner E.S., Appel A.M., Raugei S., Shaw W.J., Bullock R.M. (2022). Molecular Catalysts with Diphosphine Ligands Containing Pendant Amines. Chem. Rev..

[171] Raugei S., Chen S., Ho M.-H., Ginovska-Pangovska B., Rousseau R.J., Dupuis M., DuBois D.L., Bullock R.M. (2012). The Role of Pendant Amines in the Breaking and Forming of Molecular Hydrogen Catalyzed by Nickel Complexes. Chem. Eur. J..

[172] Wilson A.D., Newell R.H., McNevin M.J., Muckerman J.T., Rakowski DuBois M., DuBois D.L. (2006). Hydrogen Oxidation and Production Using Nickel-Based Molecular Catalysts with Positioned Proton Relays. J. Am. Chem. Soc..

[173] Franz J.A., O’Hagan M., Ho M.-H., Liu T., Helm M.L., Lense S., DuBois D.L., Shaw W.J., Appel A.M., Raugei S. (2013). Conformational Dynamics and Proton Relay Positioning in Nickel Catalysts for Hydrogen Production and Oxidation. Organometallics.

[174] Camara J.M., Rauchfuss T.B. (2012). Combining acid–base, redox and substrate binding functionalities to give a complete model for the [FeFe]-hydrogenase. Nat. Chem..

[175] Lee C.H., Dogutan D.K., Nocera D.G. (2011). Hydrogen Generation by Hangman Metalloporphyrins. J. Am. Chem. Soc..

[176] Bediako D.K., Solis B.H., Dogutan D.K., Roubelakis M.M., Maher A.G., Lee C.H., Chambers M.B., Hammes-Schiffer S., Nocera D.G. (2014). Role of pendant proton relays and proton-coupled electron transfer on the hydrogen evolution reaction by nickel hangman porphyrins. Proc. Natl. Acad. Sci. USA.

[177] Bhunia S., Rana A., Hematian S., Karlin K.D., Dey A. (2021). Proton Relay in Iron Porphyrins for Hydrogen Evolution Reaction. Inorg. Chem..

[178] Solis B.H., Maher A.G., Honda T., Powers D.C., Nocera D.G., Hammes-Schiffer S. (2014). Theoretical Analysis of Cobalt Hangman Porphyrins: Ligand Dearomatization and Mechanistic Implications for Hydrogen Evolution. ACS Catal..

[179] Kasemthaveechok S., Fabre B., Loget G., Gramage-Doria R. (2019). Remote ion-pair interactions in Fe-porphyrin-based molecular catalysts for the hydrogen evolution reaction. Catal. Sci. Technol..

[180] Fang J.-J., Lan J., Yang G., Yuan G.-Q., Liu H.-Y., Si L.-P. (2021). Synthesis of cobalt A_2_B triaryl corroles bearing aldehyde and amide pyridyl groups and their performance in electrocatalytic hydrogen evolution. New J. Chem..

[181] Zhu Z.-M., Peng W.-Y., Yang W., Ling C., Zhang H., Si L.-P., Liu H.-Y. (2022). Synthesis of cobalt A_2_B triaryl corroles bearing methoxy or hydroxyl groups and their activity in electrocatalytic hydrogen evolution. Appl. Organomet. Chem..

[182] Roubelakis M.M., Bediako D.K., Dogutan D.K., Nocera D.G. (2021). Influence of the proton relay spacer on hydrogen electrocatalysis by cobalt hangman porphyrins. J. Porphyr. Phthalocyanines.

[183] Zhou H., Groves J.T. (2004). Host-guest interactions of cyclodextrins and metalloporphyrins: Supramolecular building blocks toward artificial heme proteins. J. Porphyr. Phthalocyanines.

[184] Singleton M.L., Crouthers D.J., Duttweiler R.P.I., Reibenspies J.H., Darensbourg M.Y. (2011). Sulfonated Diiron Complexes as Water-Soluble Models of the [Fe–Fe]-Hydrogenase Enzyme Active Site. Inorg. Chem..

[185] Zaffaroni R., Orth N., Ivanović-Burmazović I., Reek J.N.H. (2020). Hydrogenase Mimics in M_12_L_24_ Nanospheres to Control Overpotential and Activity in Proton-Reduction Catalysis. Angew. Chem..

[186] Jones A.K., Lichtenstein B.R., Dutta A., Gordon G., Dutton P.L. (2007). Synthetic Hydrogenases: Incorporation of an Iron Carbonyl Thiolate into a Designed Peptide. J. Am. Chem. Soc..

[187] Apfel U.-P., Rudolph M., Apfel C., Robl C., Langenegger D., Hoyer D., Jaun B., Ebert M.-O., Alpermann T., Seebach D. (2010). Reaction of Fe_3_(CO)_12_ with octreotide—Chemical, electrochemical and biological investigations. Dalton Trans..

[188] Roy S., Shinde S., Hamilton G.A., Hartnett H.E., Jones A.K. (2011). Artificial [FeFe]-Hydrogenase: On Resin Modification of an Amino Acid to Anchor a Hexacarbonyldiiron Cluster in a Peptide Framework. Eur. J. Inorg. Chem..

[189] Sano Y., Onoda A., Hayashi T. (2012). Photocatalytic hydrogen evolution by a diiron hydrogenase model based on a peptide fragment of cytochrome c556 with an attached diiron carbonyl cluster and an attached ruthenium photosensitizer. J. Inorg. Biochem..

[190] Roy S., Nguyen T.-A.D., Gan L., Jones A.K. (2015). Biomimetic peptide-based models of [FeFe]-hydrogenases: Utilization of phosphine-containing peptides. Dalton Trans..

[191] Gloaguen F., Lawrence J.D., Rauchfuss T.B. (2001). Biomimetic Hydrogen Evolution Catalyzed by an Iron Carbonyl Thiolate. J. Am. Chem. Soc..

[192] Dutta A., Lense S., Hou J., Engelhard M.H., Roberts J.A.S., Shaw W.J. (2013). Minimal Proton Channel Enables H_2_ Oxidation and Production with a Water-Soluble Nickel-Based Catalyst. J. Am. Chem. Soc..

[193] Dutta A., Roberts J.A.S., Shaw W.J. (2014). Arginine-containing ligands enhance H₂ oxidation catalyst performance. Angew. Chem. Int. Ed. Engl..

[194] Brazzolotto D., Gennari M., Queyriaux N., Simmons T.R., Pécaut J., Demeshko S., Meyer F., Orio M., Artero V., Duboc C. (2016). Nickel-centred proton reduction catalysis in a model of [NiFe] hydrogenase. Nat. Chem..

[195] Tang H., Hall M.B. (2017). Biomimetics of [NiFe]-Hydrogenase: Nickel- or Iron-Centered Proton Reduction Catalysis?. J. Am. Chem. Soc..

[196] Dutta A., Hamilton G.A., Hartnett H.E., Jones A.K. (2012). Construction of Heterometallic Clusters in a Small Peptide Scaffold As [NiFe]-Hydrogenase Models: Development of a Synthetic Methodology. Inorg. Chem..

[197] Le J.M., Alachouzos G., Chino M., Frontier A.J., Lombardi A., Bren K.L. (2020). Tuning Mechanism through Buffer Dependence of Hydrogen Evolution Catalyzed by a Cobalt Mini-Enzyme. Biochemistry.

[198] Chino M., Leone L., Zambrano G., Pirro F., D’Alonzo D., Firpo V., Aref D., Lista L., Maglio O., Nastri F. (2018). Oxidation catalysis by iron and manganese porphyrins within enzyme-like cages. Biopolymers.

[199] Dong Y., Chen Y.-M., Kong X.-J., Gao S.-Q., Lang J.-J., Du K.-J., Lin Y.-W. (2022). Rational design of an artificial hydrolytic nuclease by introduction of a sodium copper chlorophyllin in L29E myoglobin. J. Inorg. Biochem..

[200] Marchi-Delapierre C., Rondot L., Cavazza C., Ménage S. (2015). Oxidation Catalysis by Rationally Designed Artificial Metalloenzymes. Isr. J. Chem..

[201] Edwards E.H., Bren K.L. (2020). Light-driven catalysis with engineered enzymes and biomimetic systems. Biotechnol. Appl. Biochem..

[202] Pal U., Ghosh S., Chatterjee D. (2012). Effect of sacrificial electron donors on hydrogen generation over visible light—Irradiated nonmetal-doped TiO2 photocatalysts. Transit. Met. Chem..

[203] Pellegrin Y., Odobel F. (2017). Sacrificial electron donor reagents for solar fuel production. Comptes Rendus Chim..

[204] Ge P., Olaya A.J., Scanlon M.D., Hatay Patir I., Vrubel H., Girault H.H. (2013). Photoinduced Biphasic Hydrogen Evolution: Decamethylosmocene As a Light-Driven Electron Donor. ChemPhysChem.

[205] Ge P., Hojeij M., Scanlon M.D., Girault H.H. (2020). Photo-Recycling the Sacrificial Electron Donor: Towards Sustainable Hydrogen Evolution in a Biphasic System. ChemPhysChem.

[206] Gao M., Zhu L., Ong W.L., Wang J., Ho G.W. (2015). Structural design of TiO_2_-based photocatalyst for H_2_ production and degradation applications. Catal. Sci. Technol..

[207] Xu F., Shen Y., Sun L., Zeng H., Lu Y. (2011). Enhanced photocatalytic activity of hierarchical ZnO nanoplate-nanowire architecture as environmentally safe and facilely recyclable photocatalyst. Nanoscale.

[208] Zhou X., Xu Q., Lei W., Zhang T., Qi X., Liu G., Deng K., Yu J. (2014). Origin of Tunable Photocatalytic Selectivity of Well-Defined α-Fe_2_O_3_ Nanocrystals. Small.

[209] Shang L., Tong B., Yu H., Waterhouse G.I.N., Zhou C., Zhao Y., Tahir M., Wu L.-Z., Tung C.-H., Zhang T. (2016). CdS Nanoparticle-Decorated Cd Nanosheets for Efficient Visible Light-Driven Photocatalytic Hydrogen Evolution. Adv. Energy Mater..

[210] Jayakumar J., Chou H.-H. (2020). Recent Advances in Visible-Light-Driven Hydrogen Evolution from Water Using Polymer Photocatalysts. ChemCatChem.

[211] Ong W.-J., Tan L.-L., Ng Y.H., Yong S.-T., Chai S.-P. (2016). Graphitic Carbon Nitride (g-C3N4)-Based Photocatalysts for Artificial Photosynthesis and Environmental Remediation: Are We a Step Closer to Achieving Sustainability?. Chem. Rev..

[212] Banerjee T., Gottschling K., Savasci G., Ochsenfeld C., Lotsch B.V. (2018). H_2_ Evolution with Covalent Organic Framework Photocatalysts. ACS Energy Lett..

[213] Li C., Liu J., Li H., Wu K., Wang J., Yang Q. (2022). Covalent organic frameworks with high quantum efficiency in sacrificial photocatalytic hydrogen evolution. Nat. Commun..

[214] Zhao Z., Zheng Y., Wang C., Zhang S., Song J., Li Y., Ma S., Cheng P., Zhang Z., Chen Y. (2021). Fabrication of Robust Covalent Organic Frameworks for Enhanced Visible-Light-Driven H_2_ Evolution. ACS Catal..

[215] Ghosh S., Nakada A., Springer M.A., Kawaguchi T., Suzuki K., Kaji H., Baburin I., Kuc A., Heine T., Suzuki H. (2020). Identification of Prime Factors to Maximize the Photocatalytic Hydrogen Evolution of Covalent Organic Frameworks. J. Am. Chem. Soc..

[216] Hisatomi T., Domen K. (2019). Reaction systems for solar hydrogen production via water splitting with particulate semiconductor photocatalysts. Nat. Catal..

[217] Partho A.T., Tahir M., Tahir B. (2022). Recent advances in covalent organic framework (COF) nanotextures with band engineering for stimulating solar hydrogen production: A comprehensive review. Int. J. Hydrog. Energy.

[218] Zhang P., Wang M., Na Y., Li X., Jiang Y., Sun L. (2010). Homogeneous photocatalytic production of hydrogen from water by a bioinspired [Fe_2_S_2_] catalyst with high turnover numbers. Dalton Trans..

[219] Streich D., Astuti Y., Orlandi M., Schwartz L., Lomoth R., Hammarström L., Ott S. (2010). High-Turnover Photochemical Hydrogen Production Catalyzed by a Model Complex of the [FeFe]-Hydrogenase Active Site. Chem. Eur. J..

[220] Wang X.-B., Zheng H.-Q., Rao H., Yao H.-C., Fan Y.-T., Hou H.-W. (2016). Synthesis of a new iron—Sulfur cluster compound and its photocatalytic H_2_ evolution activity through visible light irradiation. Appl. Organomet. Chem..

[221] Jian J.-X., Ye C., Wang X.-Z., Wen M., Li Z.-J., Li X.-B., Chen B., Tung C.-H., Wu L.-Z. (2016). Comparison of H_2_ photogeneration by [FeFe]-hydrogenase mimics with CdSe QDs and Ru(bpy)_3_Cl_2_ in aqueous solution. Energy Environ. Sci..

[222] Goy R., Bertini L., Rudolph T., Lin S., Schulz M., Zampella G., Dietzek B., Schacher F.H., De Gioia L., Sakai K. (2017). Photocatalytic Hydrogen Evolution Driven by [FeFe] Hydrogenase Models Tethered to Fluorene and Silafluorene Sensitizers. Chem. Eur. J..

[223] McLaughlin M.P., McCormick T.M., Eisenberg R., Holland P.L. (2011). A stable molecular nickel catalyst for the homogeneous photogeneration of hydrogen in aqueous solution. Chem. Commun..

[224] Han Z., Shen L., Brennessel W.W., Holland P.L., Eisenberg R. (2013). Nickel Pyridinethiolate Complexes as Catalysts for the Light-Driven Production of Hydrogen from Aqueous Solutions in Noble-Metal-Free Systems. J. Am. Chem. Soc..

[225] Fihri A., Artero V., Pereira A., Fontecave M. (2008). Efficient H_2_-producing photocatalytic systems based on cyclometalated iridium- and tricarbonylrhenium-diimine photosensitizers and cobaloxime catalysts. Dalton Trans..

[226] Probst B., Rodenberg A., Guttentag M., Hamm P., Alberto R. (2010). A Highly Stable Rhenium—Cobalt System for Photocatalytic H2 Production: Unraveling the Performance-Limiting Steps. Inorg. Chem..

[227] Du P., Schneider J., Luo G., Brennessel W.W., Eisenberg R. (2009). Visible Light-Driven Hydrogen Production from Aqueous Protons Catalyzed by Molecular Cobaloxime Catalysts. Inorg. Chem..

[228] Natali M., Luisa A., Iengo E., Scandola F. (2014). Efficient photocatalytic hydrogen generation from water by a cationic cobalt(II) porphyrin. Chem. Commun..

[229] Beyene B.B., Hung C.-H. (2018). Photocatalytic hydrogen evolution from neutral aqueous solution by a water-soluble cobalt(II) porphyrin. Sustain. Energy Fuels.

[230] Chen W., Li S., Li X., Zhang C., Hu X., Zhu F., Shen G., Feng F. (2019). Iron sulfur clusters in protein nanocages for photocatalytic hydrogen generation in acidic aqueous solutions. Chem. Sci..

[231] Li S., Chen W., Hu X., Feng F. (2020). Self-Assembly of Albumin and [FeFe]-Hydrogenase Mimics for Photocatalytic Hydrogen Evolution. ACS Appl. Bio Mater..

[232] Sano Y., Onoda A., Hayashi T. (2011). A hydrogenase model system based on the sequence of cytochrome c: Photochemical hydrogen evolution in aqueous media. Chem. Commun..

[233] Roy A., Vaughn M.D., Tomlin J., Booher G.J., Kodis G., Simmons C.R., Allen J.P., Ghirlanda G. (2020). Enhanced Photocatalytic Hydrogen Production by Hybrid Streptavidin-Diiron Catalysts. Chem. Eur. J..

[234] Keller S.G., Probst B., Heinisch T., Alberto R., Ward T.R. (2018). Photo-Driven Hydrogen Evolution by an Artificial Hydrogenase Utilizing the Biotin-Streptavidin Technology. Helv. Chim. Acta.

[235] Sommer D.J., Vaughn M.D., Ghirlanda G. (2014). Protein secondary-shell interactions enhance the photoinduced hydrogen production of cobalt protoporphyrin IX. Chem. Commun..

[236] Sommer D.J., Vaughn M.D., Clark B.C., Tomlin J., Roy A., Ghirlanda G. (2016). Reengineering cyt b_562_ for hydrogen production: A facile route to artificial hydrogenases. Biochim. Biophys. Acta BBA Bioenerg..

[237] Udry G.A.O., Tiessler-Sala L., Pugliese E., Urvoas A., Halime Z., Maréchal J.-D., Mahy J.-P., Ricoux R. (2022). Photocatalytic Hydrogen Production and Carbon Dioxide Reduction Catalyzed by an Artificial Cobalt Hemoprotein. Int. J. Mol. Sci..

[238] Edwards E.H., Jelušić J., Chakraborty S., Bren K.L. (2021). Photochemical hydrogen evolution from cobalt microperoxidase-11. J. Inorg. Biochem..

[239] Edwards E.H., Le J.M., Salamatian A.A., Peluso N.L., Leone L., Lombardi A., Bren K.L. (2022). A cobalt mimochrome for photochemical hydrogen evolution from neutral water. J. Inorg. Biochem..

[240] Utschig L.M., Silver S.C., Mulfort K.L., Tiede D.M. (2011). Nature-Driven Photochemistry for Catalytic Solar Hydrogen Production: A Photosystem I—Transition Metal Catalyst Hybrid. J. Am. Chem. Soc..

[241] Silver S.C., Niklas J., Du P., Poluektov O.G., Tiede D.M., Utschig L.M. (2013). Protein Delivery of a Ni Catalyst to Photosystem I for Light-Driven Hydrogen Production. J. Am. Chem. Soc..

[242] Soltau S.R., Niklas J., Dahlberg P.D., Poluektov O.G., Tiede D.M., Mulfort K.L., Utschig L.M. (2015). Aqueous light driven hydrogen production by a Ru–ferredoxin–Co biohybrid. Chem. Commun..

[243] Stevenson M.J., Marguet S.C., Schneider C.R., Shafaat H.S. (2017). Light-Driven Hydrogen Evolution by Nickel-Substituted Rubredoxin. ChemSusChem.

[244] Malayam Parambath S., Williams A.E., Hunt L.A., Selvan D., Hammer N.I., Chakraborty S. (2021). A De Novo-Designed Artificial Metallopeptide Hydrogenase: Insights into Photochemical Processes and the Role of Protonated Cys. ChemSusChem.

[245] Prasad P., Hunt L.A., Pall A.E., Ranasinghe M., Williams A.E., Stemmler T.L., Demeler B., Hammer N.I., Chakraborty S. (2023). Photocatalytic Hydrogen Evolution by a De Novo Designed Metalloprotein that Undergoes Ni-Mediated Oligomerization Shift. Chem. Eur. J..

[246] Deponti E., Natali M. (2016). Photocatalytic hydrogen evolution with ruthenium polypyridine sensitizers: Unveiling the key factors to improve efficiencies. Dalton Trans..

[247] Wang Y., Zhao X., Zhao Y., Yang T., Liu X., Xie J., Li G., Zhu D., Tan H., Su Z. (2019). Photosensitizers based on Ir(III) complexes for highly efficient photocatalytic hydrogen generation. Dyes Pigments.

[248] Mou Z., Dong Y., Li S., Du Y., Wang X., Yang P., Wang S. (2011). Eosin Y functionalized graphene for photocatalytic hydrogen production from water. Int. J. Hydrog. Energy.

[249] Majek M., Filace F., von Wangelin A.J. (2014). On the mechanism of photocatalytic reactions with eosin Y. Beilstein J. Org. Chem..

[250] Gimbert-Suriñach C., Albero J., Stoll T., Fortage J., Collomb M.-N., Deronzier A., Palomares E., Llobet A. (2014). Efficient and Limiting Reactions in Aqueous Light-Induced Hydrogen Evolution Systems using Molecular Catalysts and Quantum Dots. J. Am. Chem. Soc..

[251] Fan X.-B., Yu S., Hou B., Kim J.M. (2019). Quantum Dots Based Photocatalytic Hydrogen Evolution. Isr. J. Chem..

[252] Nozik A.J., Beard M.C., Luther J.M., Law M., Ellingson R.J., Johnson J.C. (2010). Semiconductor Quantum Dots and Quantum Dot Arrays and Applications of Multiple Exciton Generation to Third-Generation Photovoltaic Solar Cells. Chem. Rev..

[253] Harris R.D., Bettis Homan S., Kodaimati M., He C., Nepomnyashchii A.B., Swenson N.K., Lian S., Calzada R., Weiss E.A. (2016). Electronic Processes within Quantum Dot-Molecule Complexes. Chem. Rev..

[254] Zhao J., Holmes M.A., Osterloh F.E. (2013). Quantum Confinement Controls Photocatalysis: A Free Energy Analysis for Photocatalytic Proton Reduction at CdSe Nanocrystals. ACS Nano.

[255] Eren G.O., Sadeghi S., Bahmani Jalali H., Ritter M., Han M., Baylam I., Melikov R., Onal A., Oz F., Sahin M. (2021). Cadmium-Free and Efficient Type-II InP/ZnO/ZnS Quantum Dots and Their Application for LEDs. ACS Appl. Mater. Interfaces.

[256] Xu G., Zeng S., Zhang B., Swihart M.T., Yong K.-T., Prasad P.N. (2016). New Generation Cadmium-Free Quantum Dots for Biophotonics and Nanomedicine. Chem. Rev..

[257] Prasad P., Selvan D., Chakraborty S. (2020). Biosynthetic Approaches towards the Design of Artificial Hydrogen-Evolution Catalysts. Chem. Eur. J..

[258] Utschig L.M., Soltau S.R., Tiede D.M. (2015). Light-driven hydrogen production from Photosystem I-catalyst hybrids. Curr. Opin. Chem. Biol..

[259] Lubner C.E., Grimme R., Bryant D.A., Golbeck J.H. (2010). Wiring Photosystem I for Direct Solar Hydrogen Production. Biochemistry.

[260] Cao W.-N., Wang F., Wang H.-Y., Chen B., Feng K., Tung C.-H., Wu L.-Z. (2012). Photocatalytic hydrogen production from a simple water-soluble [FeFe]-hydrogenase model system. Chem. Commun..

[261] Li X., Wang M., Chen L., Wang X., Dong J., Sun L. (2012). Photocatalytic Water Reduction and Study of the Formation of FeIFe0 Species in Diiron Catalyst Sytems. ChemSusChem.

[262] Wen M., Li X.-B., Jian J.-X., Wang X.-Z., Wu H.-L., Chen B., Tung C.-H., Wu L.-Z. (2016). Secondary coordination sphere accelerates hole transfer for enhanced hydrogen photogeneration from [FeFe]-hydrogenase mimic and CdSe QDs in water. Sci. Rep..

[263] Corredor J., Harankahage D., Gloaguen F., Rivero M.J., Zamkov M., Ortiz I. (2021). Influence of QD photosensitizers in the photocatalytic production of hydrogen with biomimetic [FeFe]-hydrogenase. Comparative performance of CdSe and CdTe. Chemosphere.

[264] Lin H.-M., Li J.-R., Mu C., Li A., Liu X.-F., Zhao P.-H., Li Y.-L., Jiang Z.-Q., Wu H.-K. (2019). Synthesis, characterization, and electrochemistry of monophosphine-containing diiron propane-1,2-dithiolate complexes related to the active site of [FeFe]-hydrogenases. Appl. Organomet. Chem..

[265] Kumar N., Kaur-Ghumaan S. (2022). Synthesis, Characterization and Electrochemical Studies of Bis(Monothiolato) FeFe Complexes [Fe_2_(μ-SC_6_H_4_-OMe-m)_2_(CO)_5_L] (L=CO, PCy_3_, PPh_3_). ChemistrySelect.

[266] Pandey I.K., Natarajan M., Kaur-Ghumaan S. (2015). Hydrogen generation: Aromatic dithiolate-bridged metal carbonyl complexes as hydrogenase catalytic site models. J. Inorg. Biochem..

[267] Gao S., Zhang W.-Y., Duan Q., Liang Q.-C., Jiang D.-Y., Zhao J.-X., Hou J.-H. (2017). An artificial [FeFe]-hydrogenase mimic with organic chromophore-linked thiolate bridges for the photochemical production of hydrogen. Chem. Pap..

[268] Song L.-C., Wang L.-X., Tang M.-Y., Li C.-G., Song H.-B., Hu Q.-M. (2009). Synthesis, Structure, and Photoinduced Catalysis of [FeFe]-Hydrogenase Active Site Models Covalently Linked to a Porphyrin or Metalloporphyrin Moiety. Organometallics.

[269] Samuel A.P.S., Co D.T., Stern C.L., Wasielewski M.R. (2010). Ultrafast Photodriven Intramolecular Electron Transfer from a Zinc Porphyrin to a Readily Reduced Diiron Hydrogenase Model Complex. J. Am. Chem. Soc..

[270] Liu J., Jiang W. (2012). Photoinduced hydrogen evolution in supramolecular devices with a rhenium photosensitizer linked to FeFe-hydrogenase model complexes. Dalton Trans..

[271] Cui H., Hu M., Wen H., Chai G., Ma C., Chen H., Chen C. (2012). Efficient [FeFe] hydrogenase mimic dyads covalently linking to iridium photosensitizer for photocatalytic hydrogen evolution. Dalton Trans..

[272] Goy R., Apfel U.-P., Elleouet C., Escudero D., Elstner M., Görls H., Talarmin J., Schollhammer P., González L., Weigand W. (2013). A Silicon-Heteroaromatic System as Photosensitizer for Light-Driven Hydrogen Production by Hydrogenase Mimics. Eur. J. Inorg. Chem..

[273] Dempsey J.L., Brunschwig B.S., Winkler J.R., Gray H.B. (2009). Hydrogen Evolution Catalyzed by Cobaloximes. Acc. Chem. Res..

[274] Baffert C., Artero V., Fontecave M. (2007). Cobaloximes as functional models for hydrogenases. 2. Proton electroreduction catalyzed by difluoroborylbis(dimethylglyoximato)cobalt(II) complexes in organic media. Inorg. Chem..

[275] Lazarides T., McCormick T., Du P., Luo G., Lindley B., Eisenberg R. (2009). Making Hydrogen from Water Using a Homogeneous System Without Noble Metals. J. Am. Chem. Soc..

[276] Caserta G., Chino M., Firpo V., Zambrano G., Leone L., D’Alonzo D., Nastri F., Maglio O., Pavone V., Lombardi A. (2018). Enhancement of Peroxidase Activity in Artificial Mimochrome VI Catalysts through Rational Design. ChemBioChem.

[277] Briffa J., Sinagra E., Blundell R. (2020). Heavy metal pollution in the environment and their toxicological effects on humans. Heliyon.

[278] Egorova K.S., Ananikov V.P. (2017). Toxicity of Metal Compounds: Knowledge and Myths. Organometallics.

